# Supplementary material for: Exploring the Antibacterial and Antiparasitic Activity of Phenylaminonaphthoquinones—Green Synthesis, Biological Evaluation and Computational Study
Source: Int J Mol Sci. 2024 Oct 3;25(19):10670. doi: 10.3390/ijms251910670 (PMC11870044; doi:10.3390/ijms251910670)

Exploring the antibacterial and antiparasitic activity of amino naphthoquinones. Green synthesis, biological evaluation and computational study

Sussan Lopez-Mercado [1], Cinthya Enríquez [2], Jaime A. Valderrama [3], Ricardo Pino-Rios [1,2,3], Liliana Ruiz-Vásquez [4], Lastenia Ruiz Mesia [4,5], Gabriel Vargas Arana [5*], and Julio Benites [1,2,3*]

1 Magister en Ciencias Químicas y Farmacéuticas, Facultad de Ciencias de la Salud, Universidad Arturo Prat, Casilla 121, Iquique 1100000, Chile; sulopez_@estudiantesunap.cl (S.L.-M.)
2 Doctorado en Química Medicinal, Facultad de Ciencias de la Salud, Universidad Arturo Prat, Casilla 121, Iquique 1100000, Chile; cenriquez@estudiantesunap.cl (C.E.)
3 Laboratorio de Química Medicinal, Química y Farmacia, Facultad de Ciencias de la Salud, Universidad Arturo Prat, Casilla 121, Iquique 1100000, Chile; jaimeadolfov@gmail.com (J.A.V.); rpinorios@unap.cl (R.P.-R.); juliob@unap.cl (J.B.)
4 Centro de Investigación de Recursos Naturales, Universidad Nacional de la Amazonía Peruana (UNAP), AA. HH. "Nuevo San Lorenzo", Pasaje Paujiles S/N, San Juan Bautista, Iquitos16002, Perú; liliana.ruiz@unapiquitos.edu.pe (L.R.-V); lasteniaruiz@unapiquitos.edu.pe (L.R.M)
5 Facultad de Farmacia y Bioquímica, Universidad Nacional de la Amazonía Peruana, Nina Rumi, San Juan Bautista, Iquitos 16000, Perú.
6 Laboratorio de Química de Productos Naturales, Instituto de Investigaciones de la Amazonía Peruana (IIAP), Av. Abelardo Quiñones km 2.5, Iquitos 16001, Perú. gvargas@iiap.gob.pe (G.V.A)
7 Facultad de Industrias Alimentarias, Universidad Nacional de la Amazonía Peruana, Iquitos 16002, Perú
* Correspondence: gvargas@iiap.gob.pe (G.V.A); juliob@unap.cl (J.B.); Tel.: +56-57-2252-6275 (J.B.)

Supporting Information

Table S1. SMILES representation of the compounds studied in this work.

Compound	SMILES
1	c1ccc2c(c1)C(=O)C=C(C2=O)Nc1ccccc1
2	c1ccc2c(c1)C(=O)C(=C(C2=O)Nc1ccccc1)Cl
3	c1ccc2c(c1)C(=O)C=C(C2=O)Nc1c(cccc1)C
4	c1ccc2c(c1)C(=O)C(=C(C2=O)Nc1c(cccc1)C)Cl
5	c1ccc2c(c1)C(=O)C=C(C2=O)Nc1ccc(cc1)O
6	c1ccc2c(c1)C(=O)C(=C(C2=O)Nc1ccc(cc1)O)Cl
7	c1ccc2c(c1)C(=O)C=C(C2=O)Nc1ccc(cc1)OC
8	c1ccc2c(c1)C(=O)C(=C(C2=O)Nc1ccc(cc1)OC)Cl
9	c1ccc2c(c1)C(=O)C=C(C2=O)Nc1c(ccc(c1)OC)OC
10	c1ccc2c(c1)C(=O)C(=C(C2=O)Nc1c(ccc(c1)OC)OC)Cl
11	c1ccc2c(c1)C(=O)C=C(C2=O)N(c1ccccc1)C
12	c1ccc2c(c1)C(=O)C(=C(C2=O)N(c1ccccc1)C)Cl
Chloroquine	CCN(CC)CCC[C@H](C)Nc1c2ccc(cc2ncc1)Cl

Figure S1. 2D representation of the interactions between compound 1 and residues of Clumping Factor A from Staphylococcus aureus4 (PDB ID: 1N67). Hydrogen atoms have been omitted in some cases for clarity.

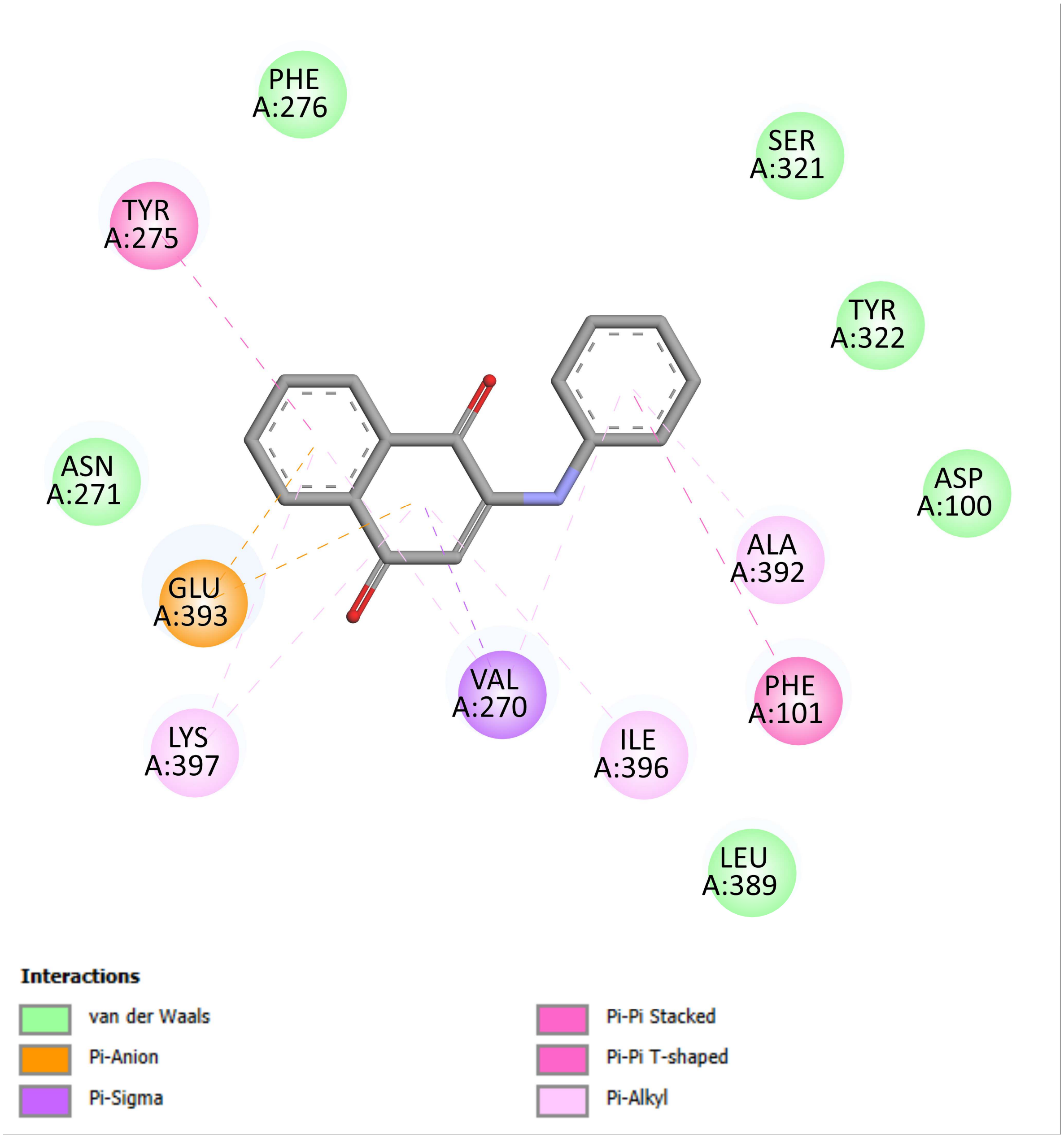

Figure S2. 2D representation of the interactions between compound 2 and residues of Clumping Factor A from Staphylococcus aureus4 (PDB ID: 1N67). Hydrogen atoms have been omitted in some cases for clarity.

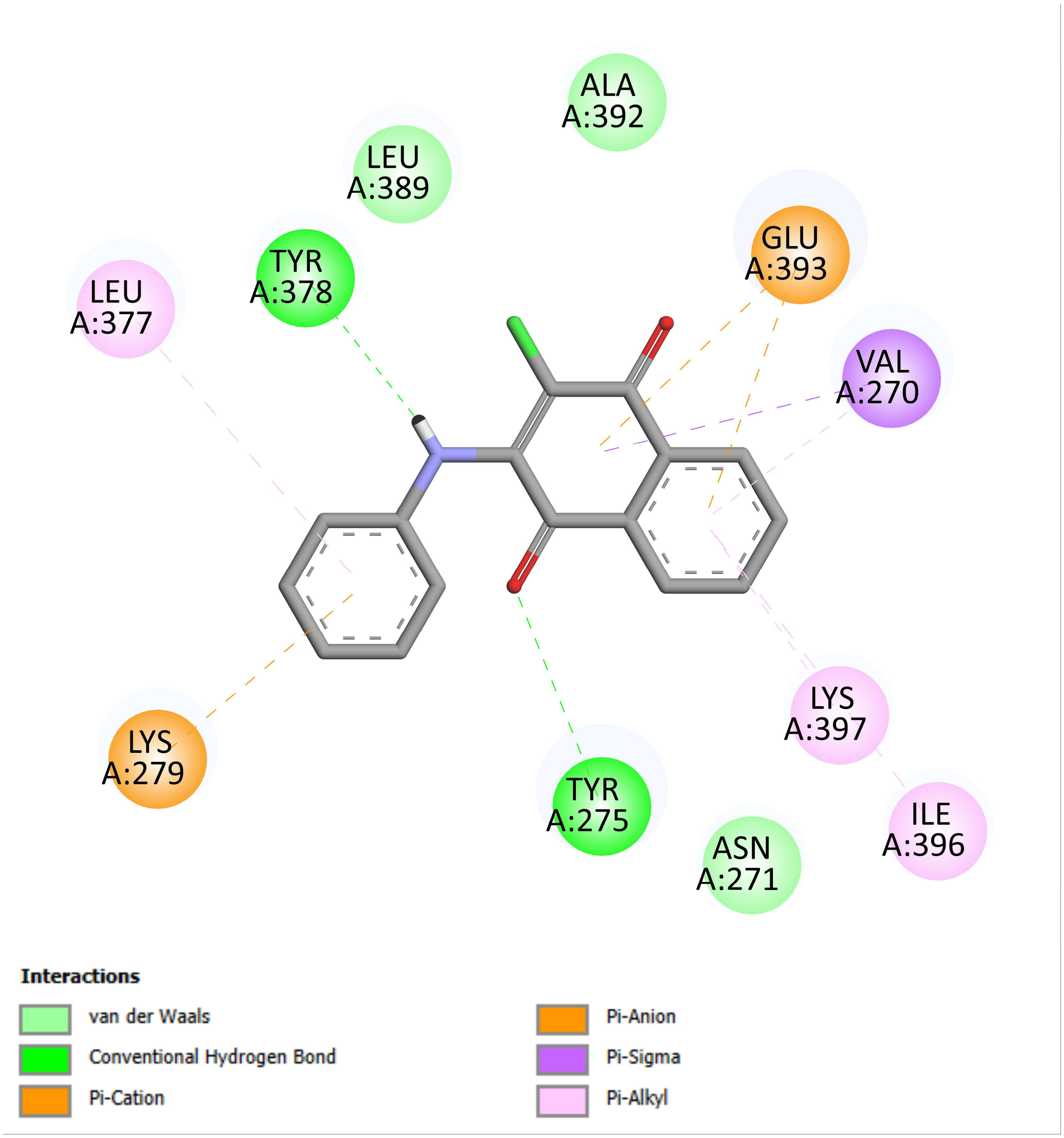

Figure S3. 2D representation of the interactions between compound 4 and residues of Clumping Factor A from Staphylococcus aureus4 (PDB ID: 1N67). Hydrogen atoms have been omitted in some cases for clarity.

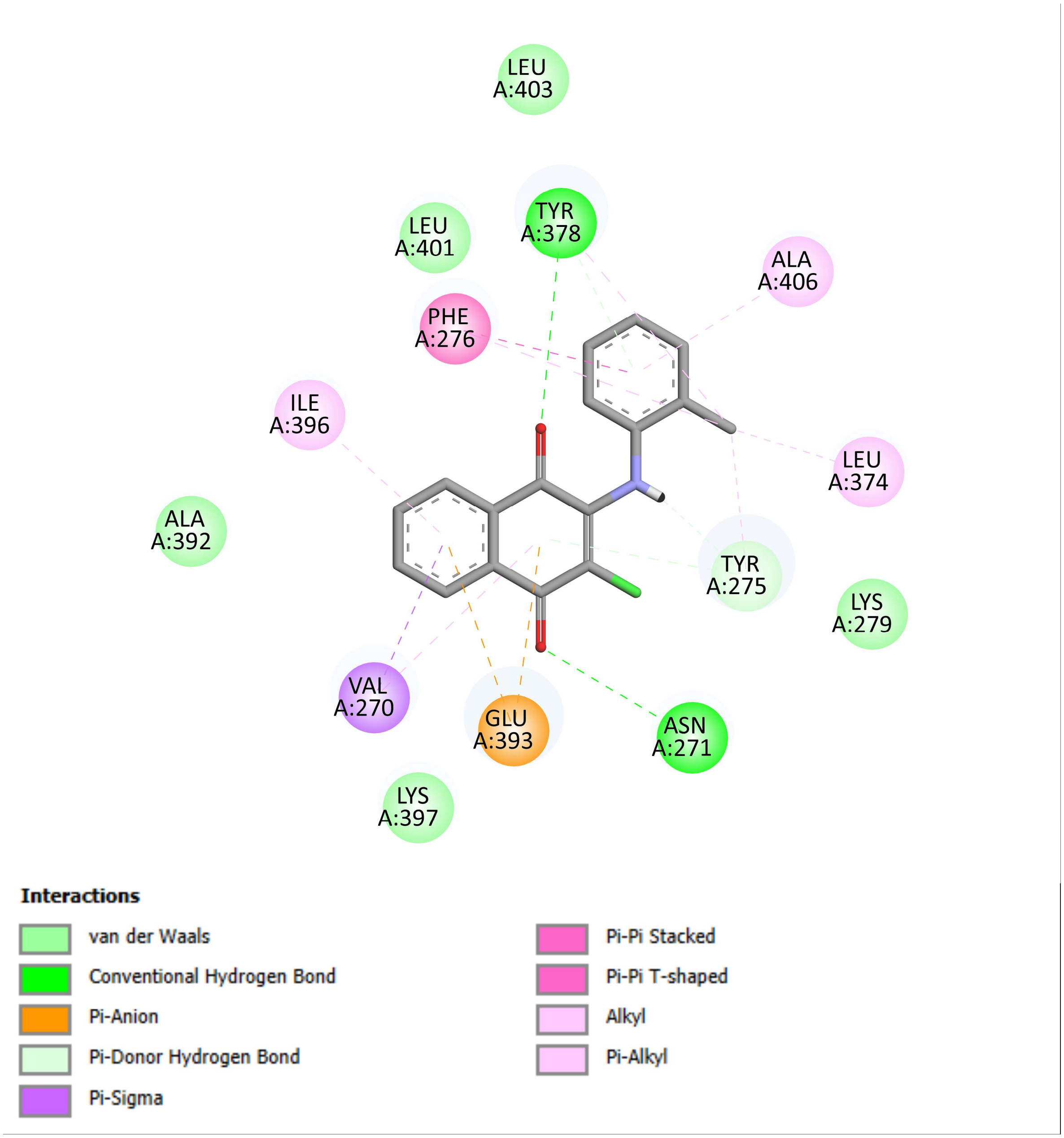

Figure S4. 2D representation of the interactions between compound 5 and residues of Clumping Factor A from Staphylococcus aureus4 (PDB ID: 1N67). Hydrogen atoms have been omitted in some cases for clarity.

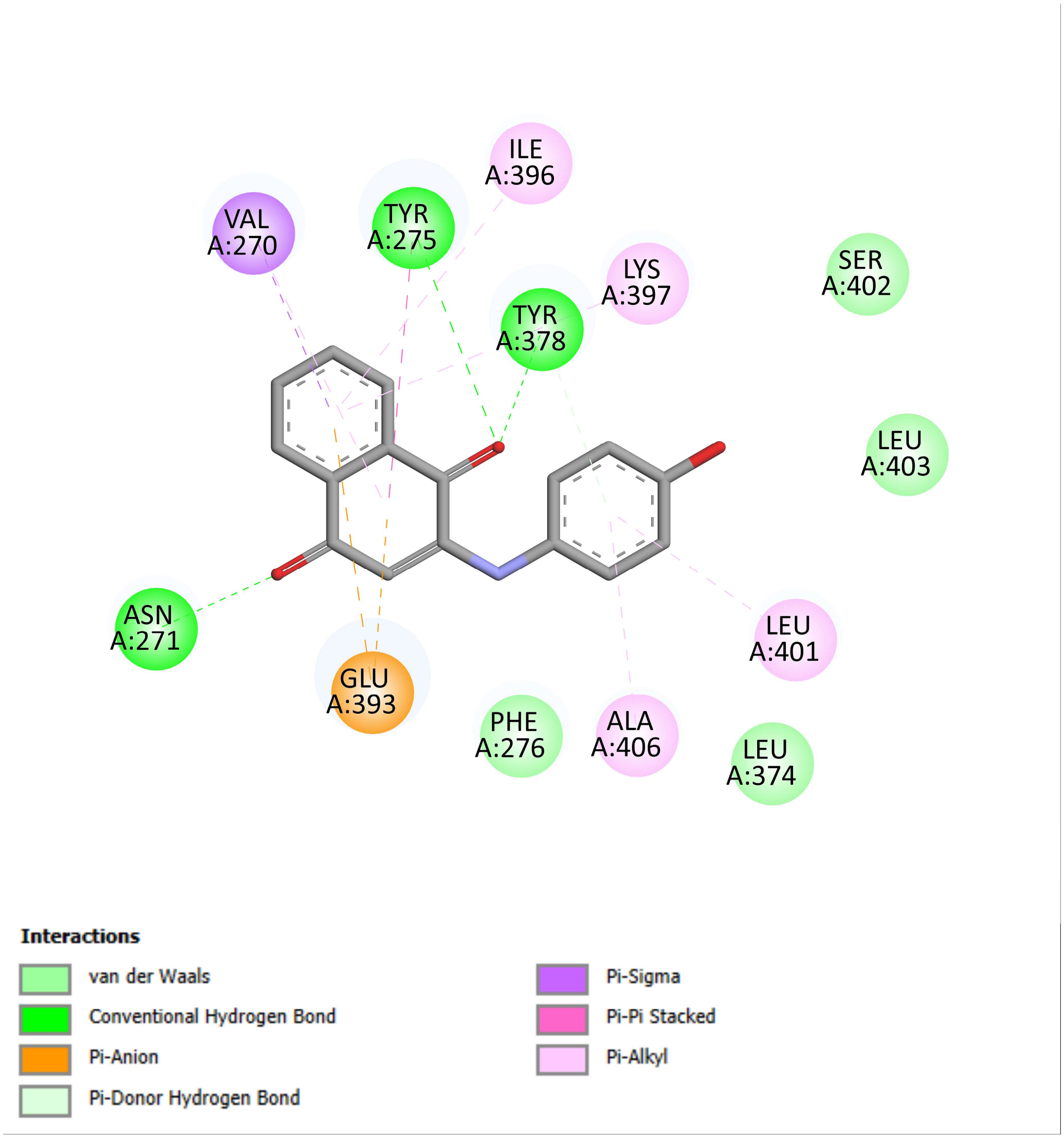

Figure S5. 2D representation of the interactions between compound 6 and residues of Clumping Factor A from Staphylococcus aureus4 (PDB ID: 1N67). Hydrogen atoms have been omitted in some cases for clarity.

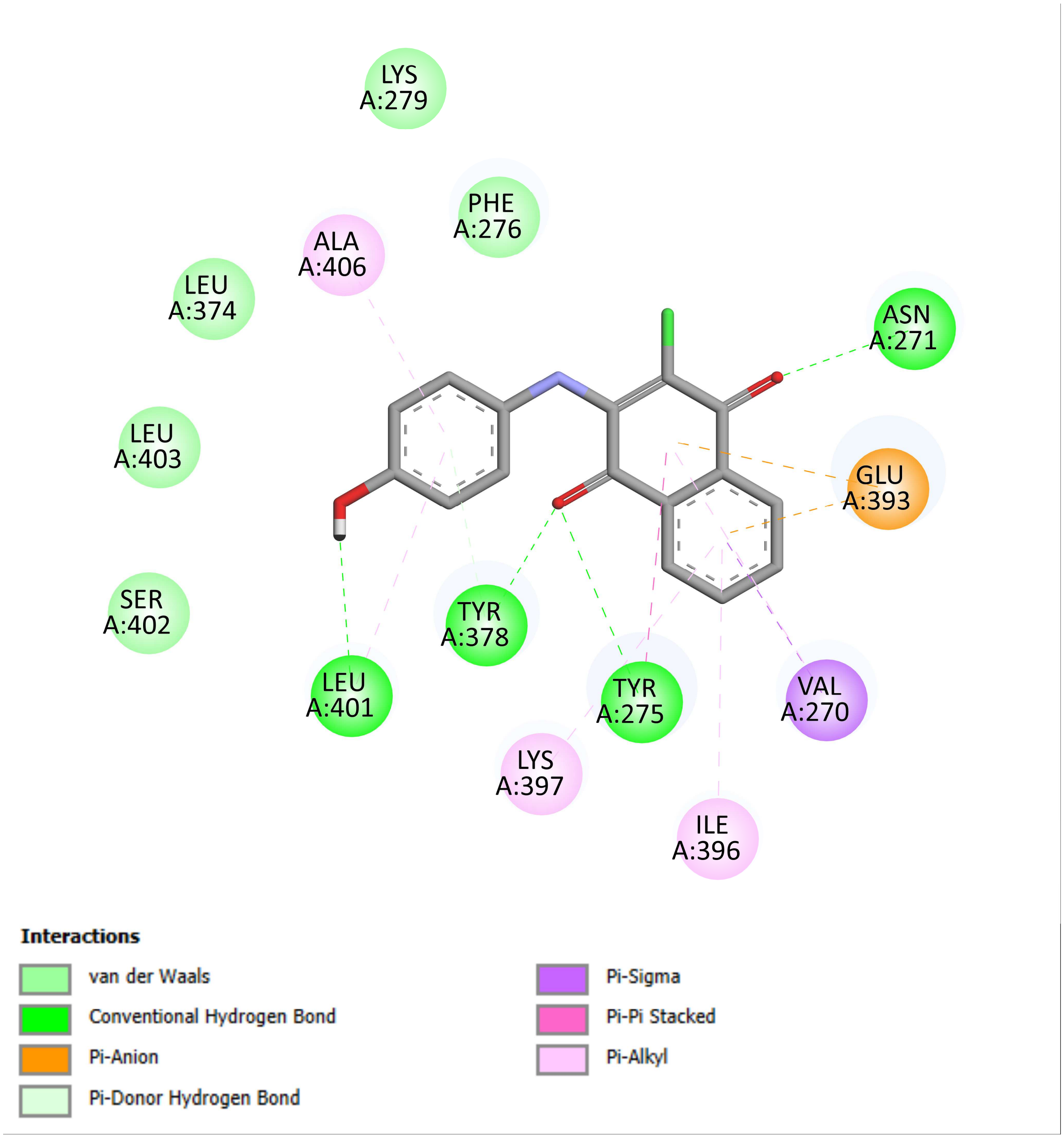

Figure S6. 2D representation of the interactions between compound 7 and residues of Clumping Factor A from Staphylococcus aureus4 (PDB ID: 1N67). Hydrogen atoms have been omitted in some cases for clarity.

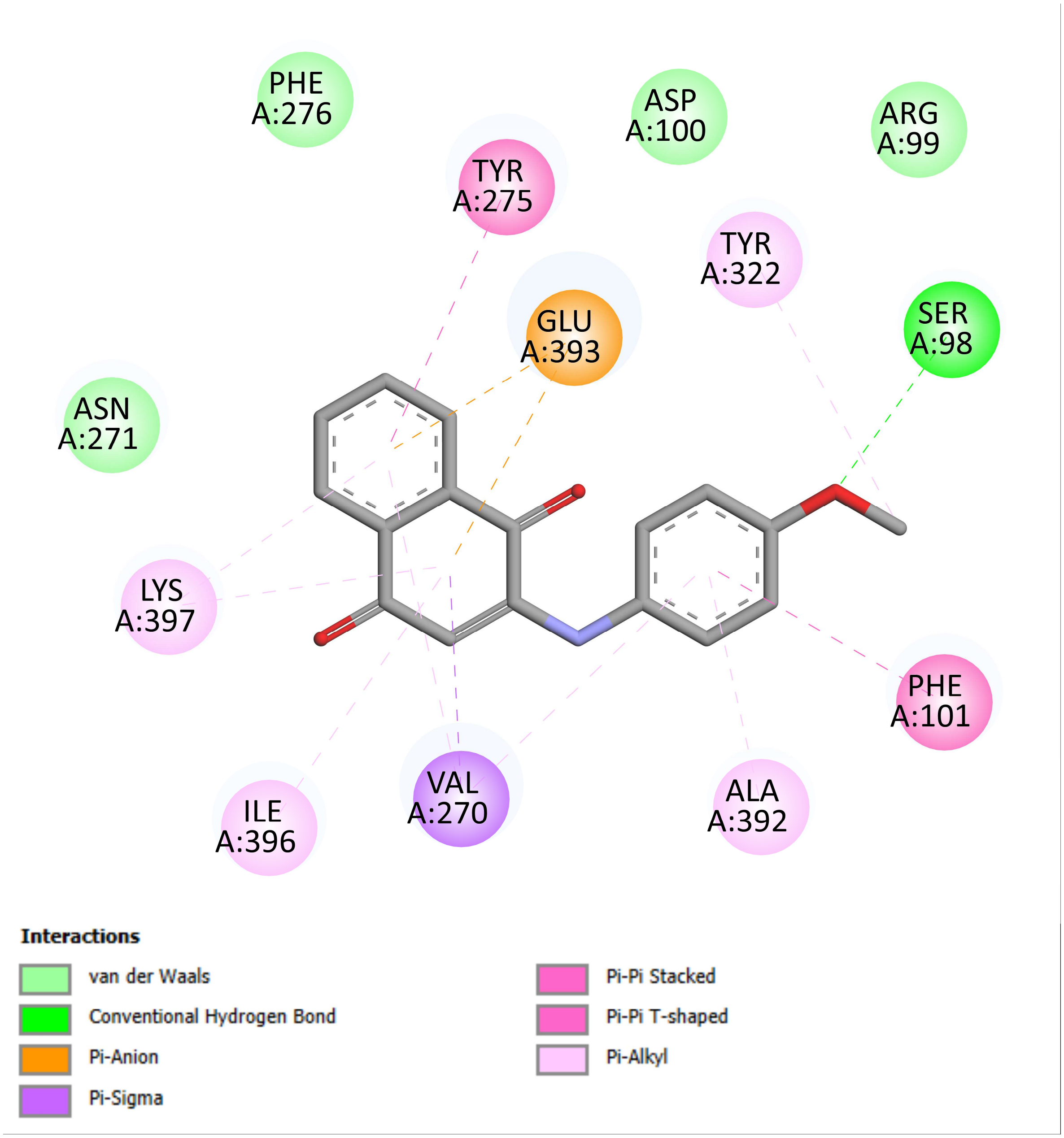

Figure S7. 2D representation of the interactions between compound 8 and residues of Clumping Factor A from Staphylococcus aureus4 (PDB ID: 1N67). Hydrogen atoms have been omitted in some cases for clarity.

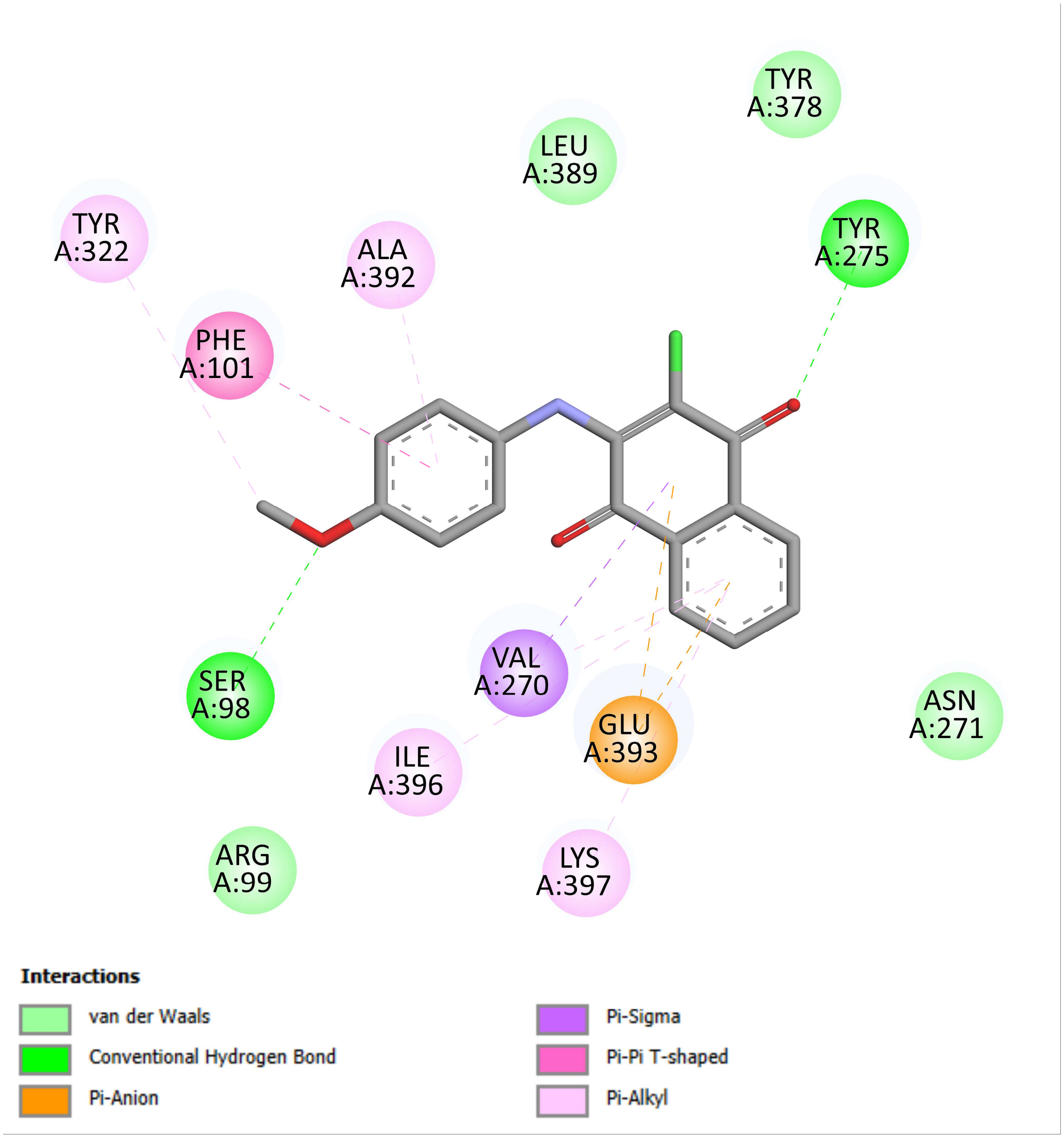

Figure S8. 2D representation of the interactions between compound 9 and residues of Clumping Factor A from Staphylococcus aureus4 (PDB ID: 1N67). Hydrogen atoms have been omitted in some cases for clarity.

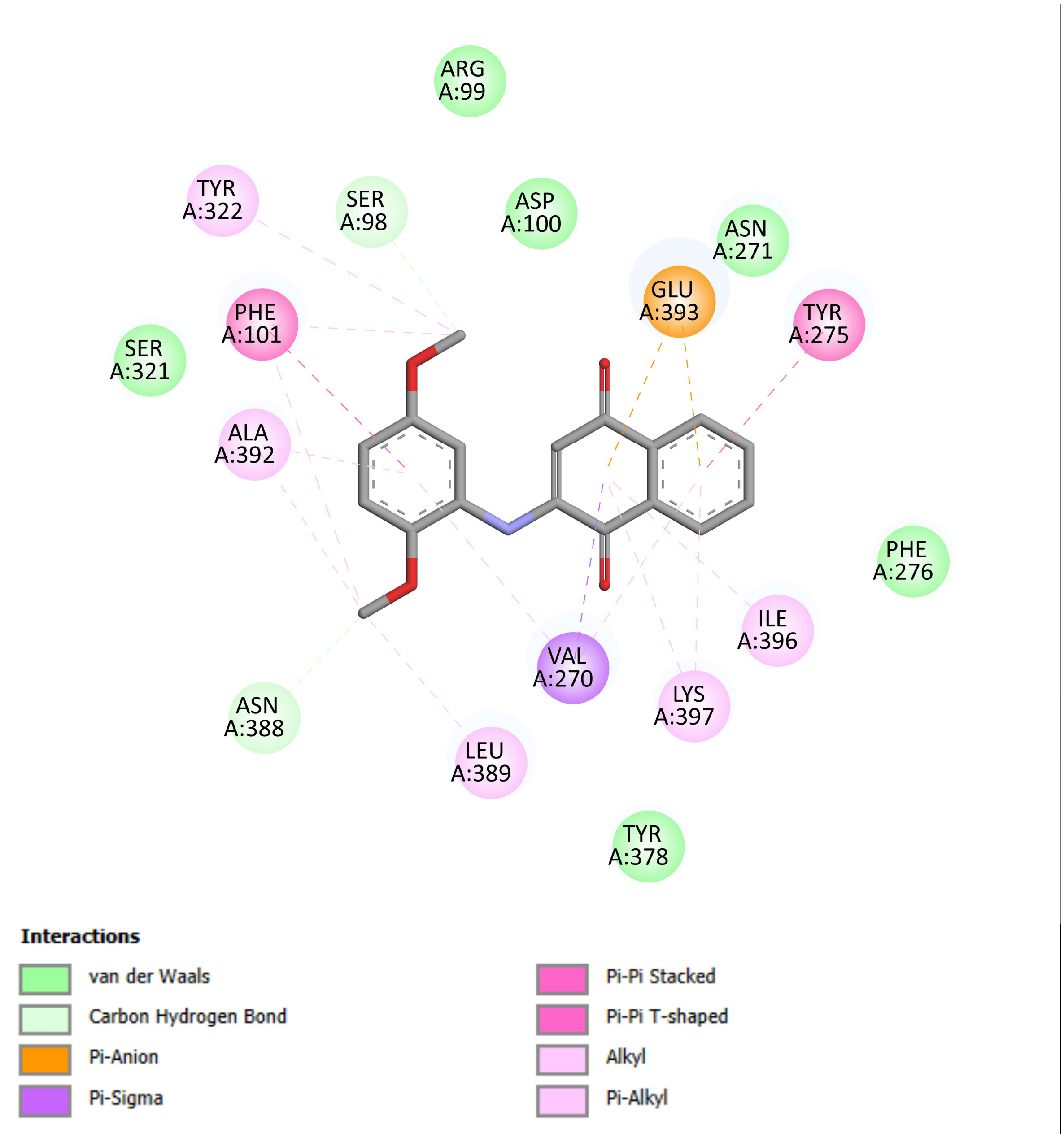

Figure S9. 2D representation of the interactions between compound 10 and residues of Clumping Factor A from Staphylococcus aureus4 (PDB ID: 1N67). Hydrogen atoms have been omitted in some cases for clarity.

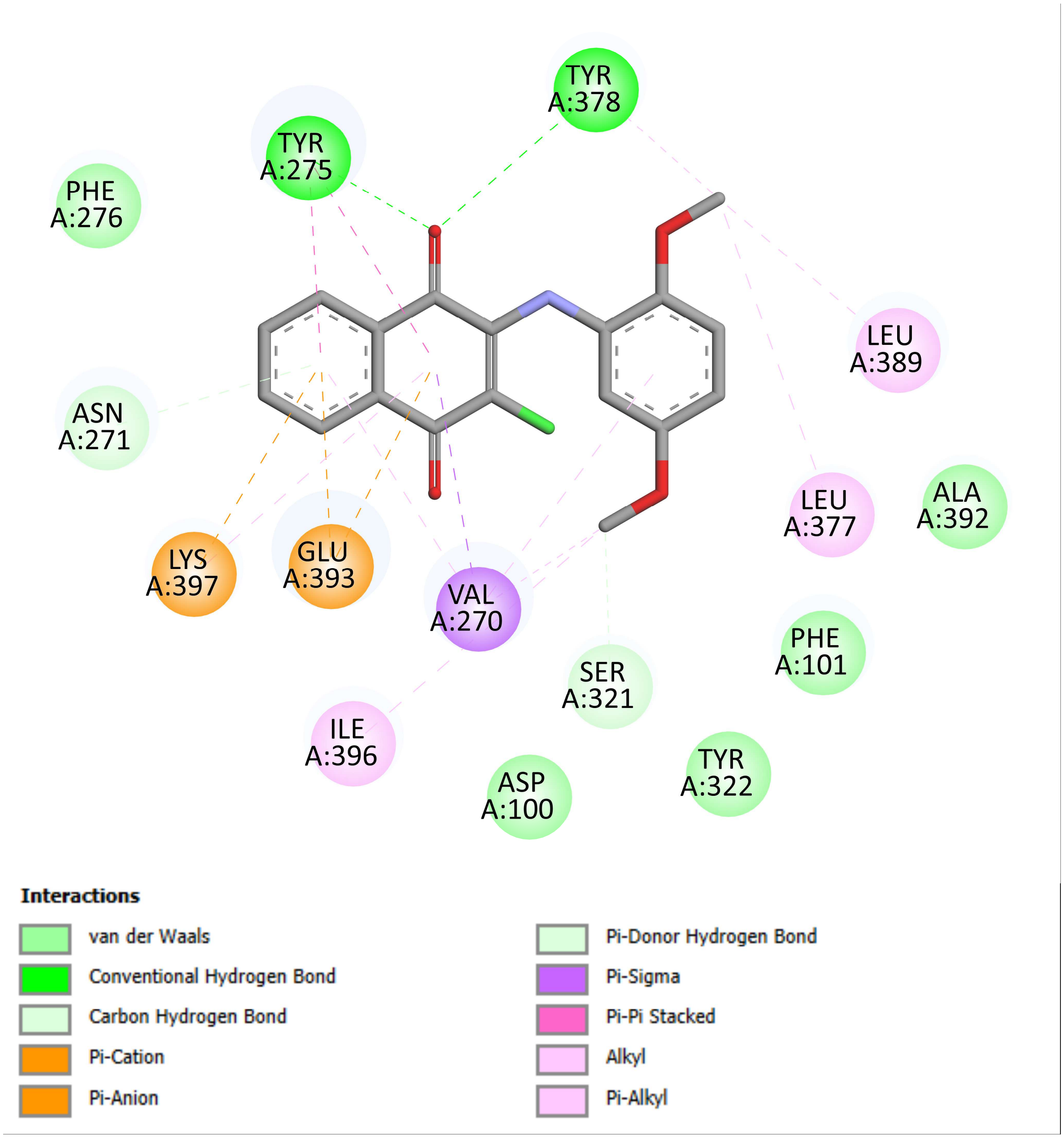

Figure S10. 2D representation of the interactions between compound 11 and residues of Clumping Factor A from Staphylococcus aureus4 (PDB ID: 1N67). Hydrogen atoms have been omitted in some cases for clarity.

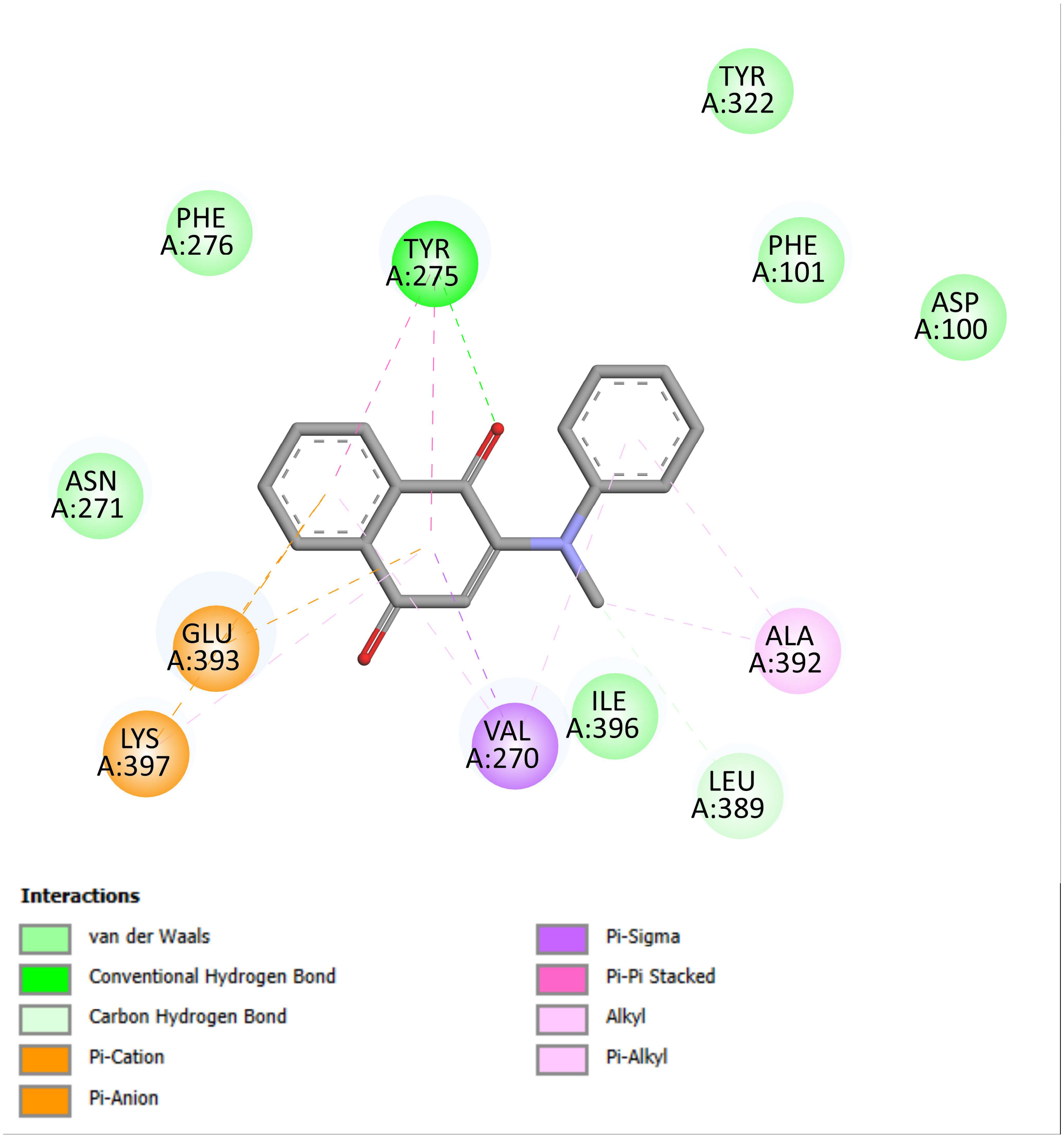

Figure S11. 2D representation of the interactions between compound 12 and residues of Clumping Factor A from Staphylococcus aureus4 (PDB ID: 1N67). Hydrogen atoms have been omitted in some cases for clarity.

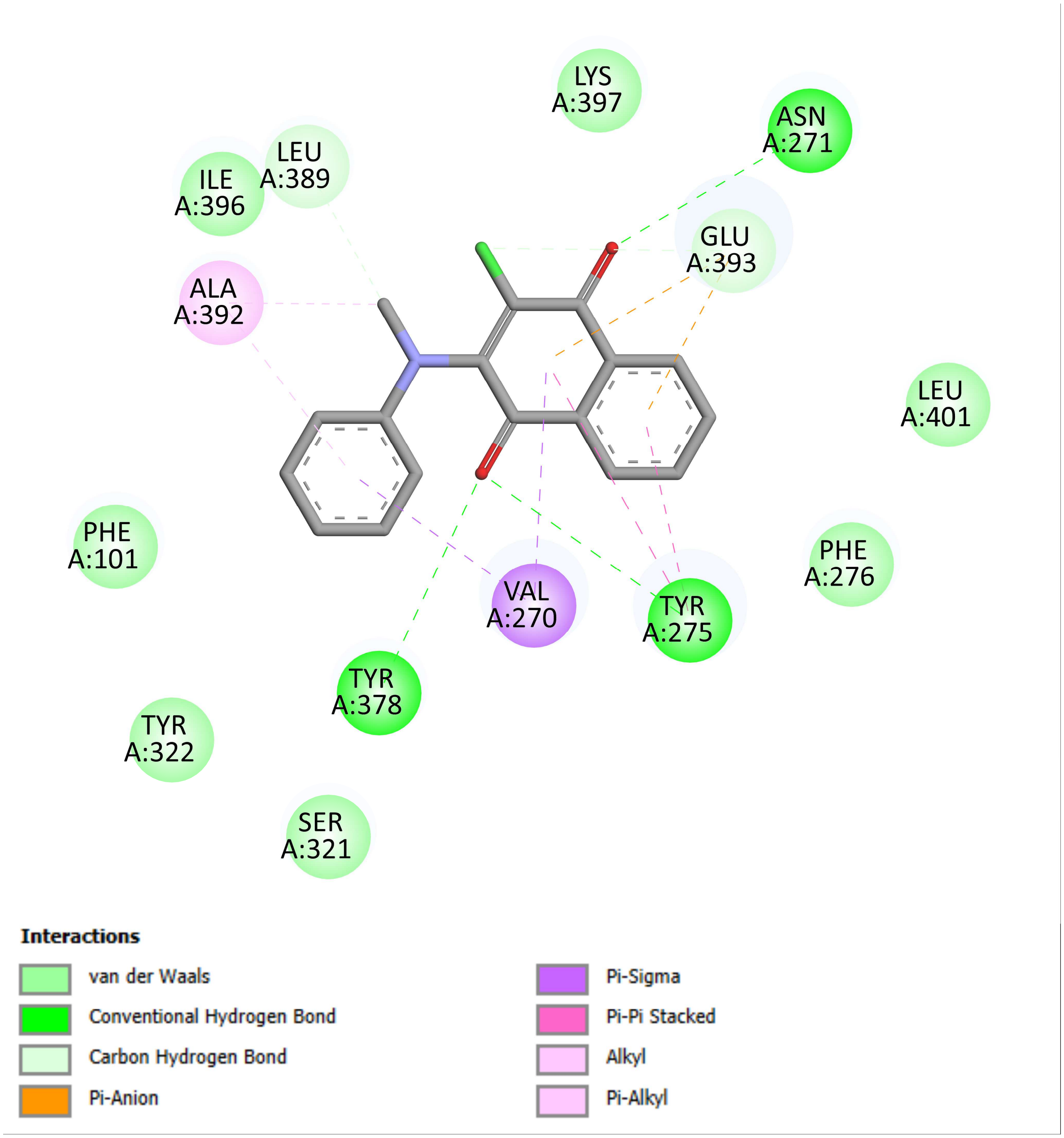

Figure S12. 2D representation of the interactions between compound 1 and residues of CYT BC1 protein (PDB ID: 4PD4). Hydrogen atoms have been omitted in some cases for clarity.

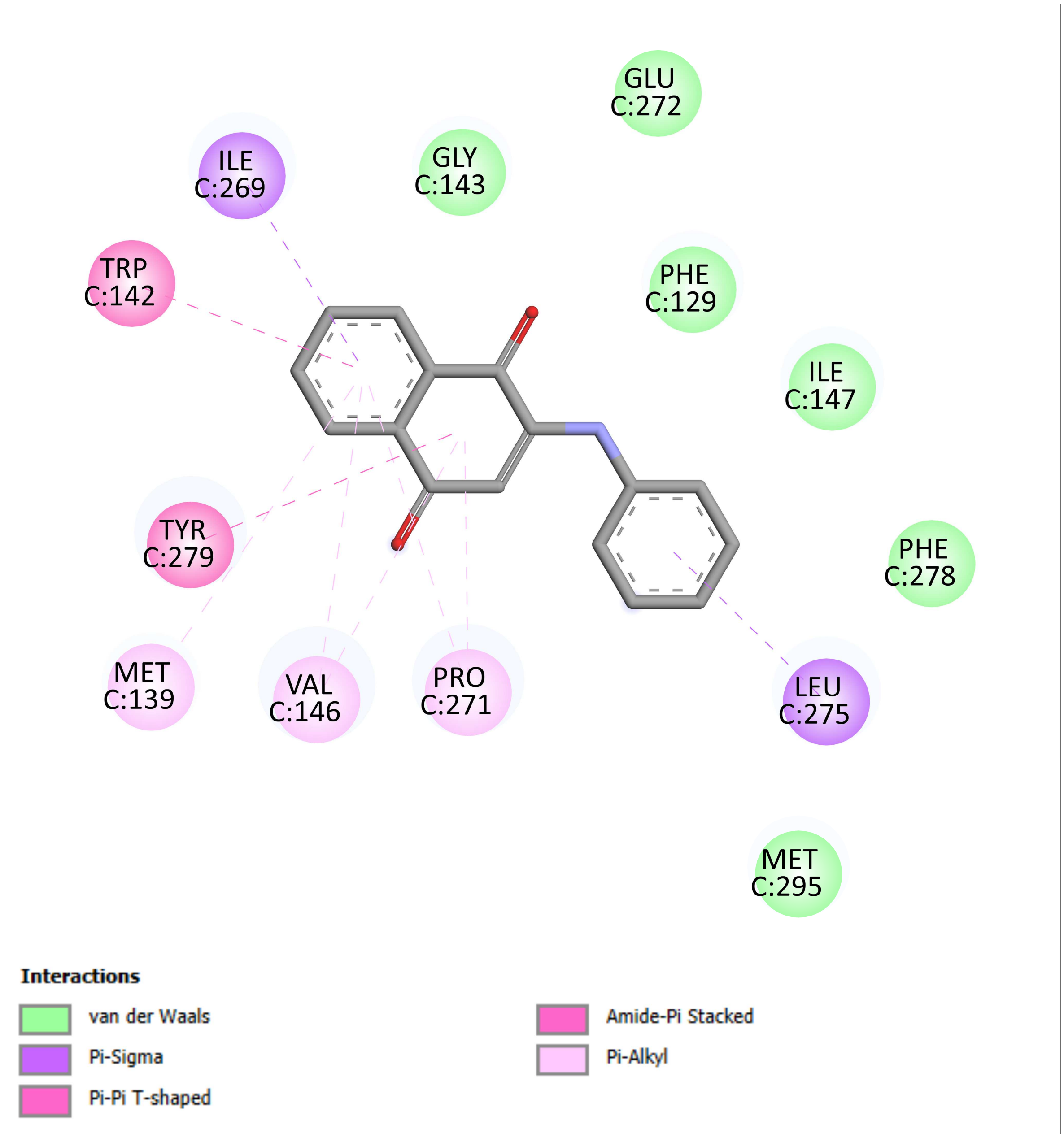

Figure S13. 2D representation of the interactions between compound 2 and residues of CYT BC1 protein (PDB ID: 4PD4). Hydrogen atoms have been omitted in some cases for clarity.

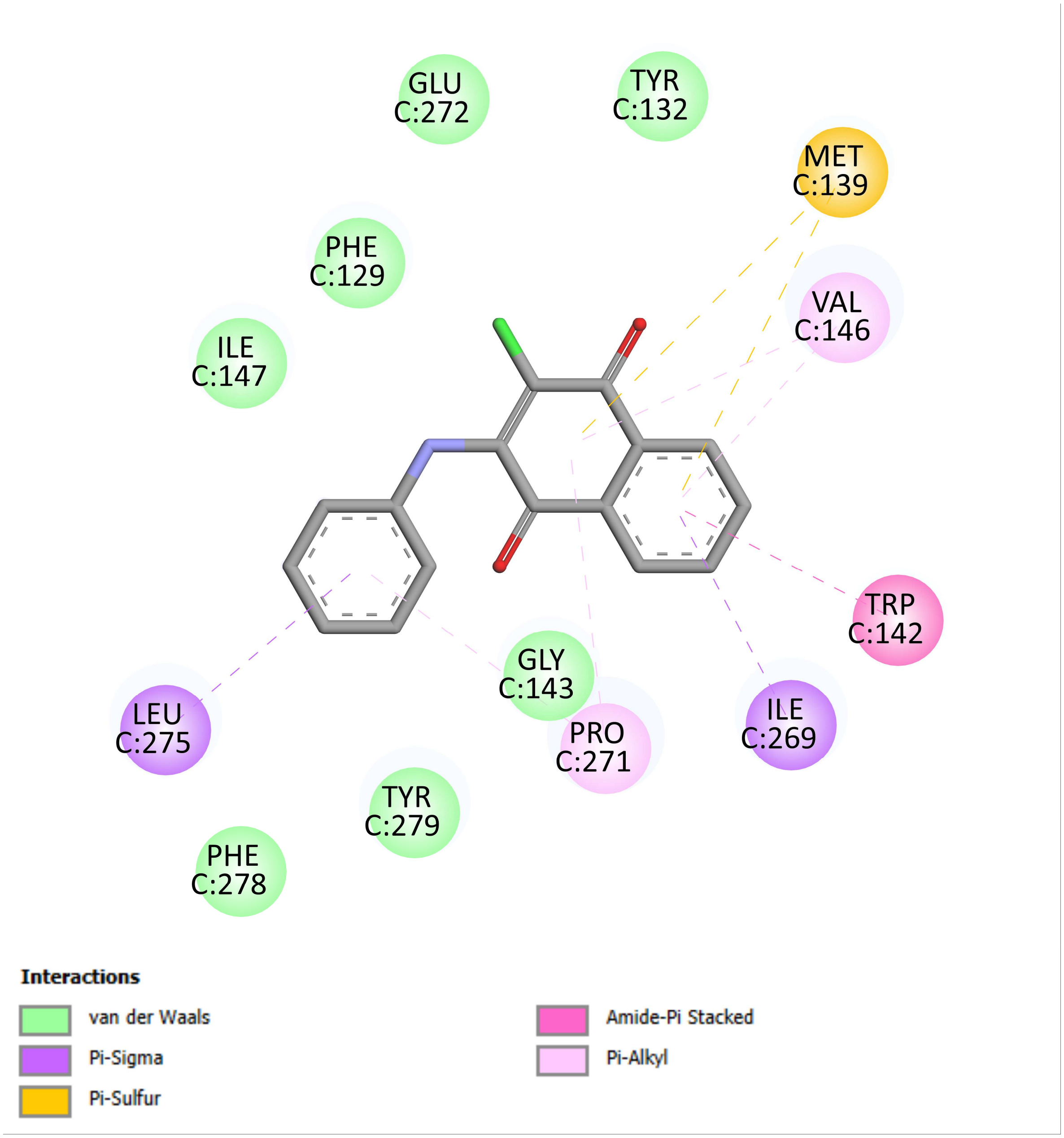

Figure S14. 2D representation of the interactions between compound 4 and residues of CYT BC1 protein (PDB ID: 4PD4). Hydrogen atoms have been omitted in some cases for clarity.

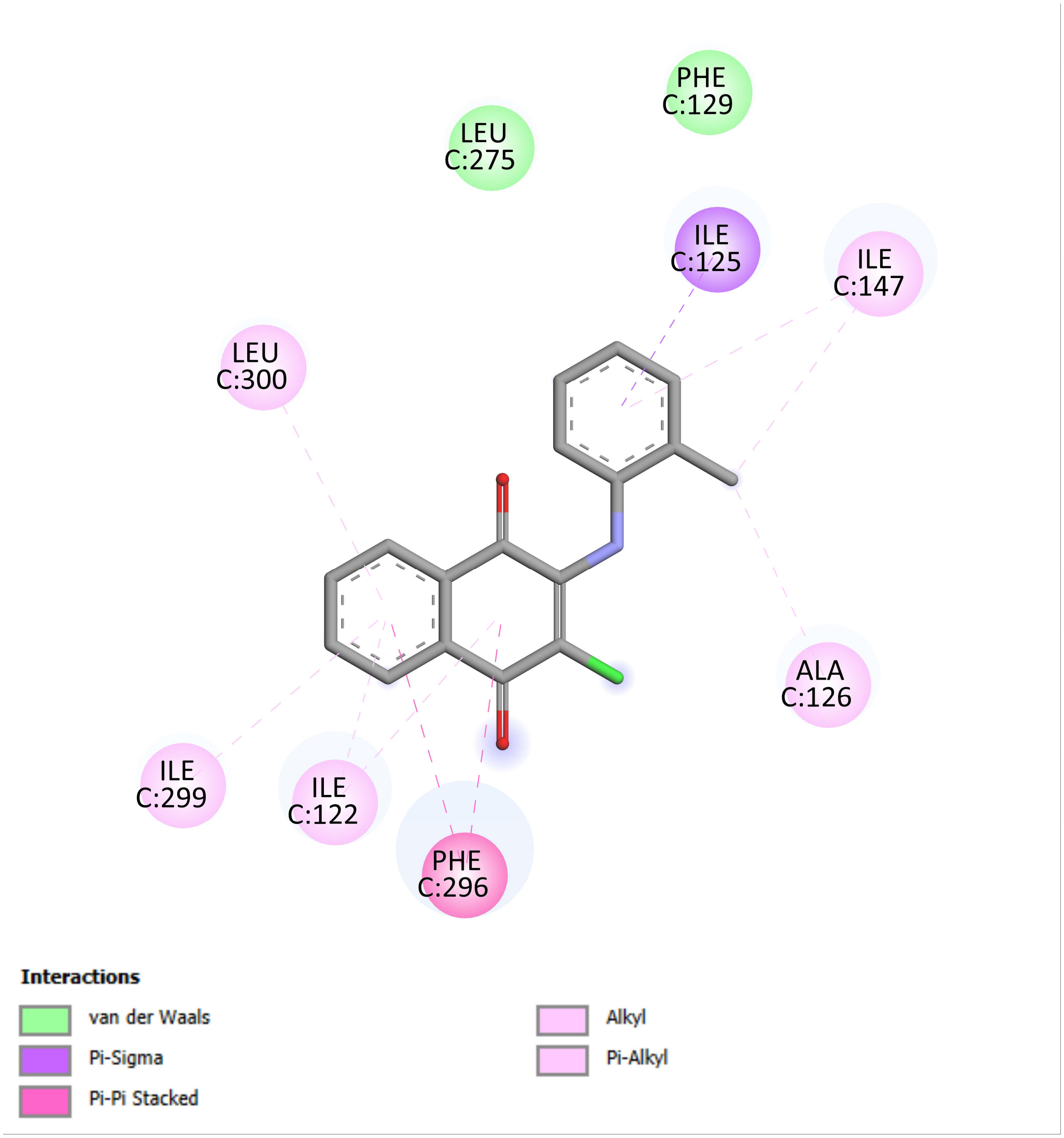

Figure S15. 2D representation of the interactions between compound 5 and residues of CYT BC1 protein (PDB ID: 4PD4). Hydrogen atoms have been omitted in some cases for clarity.

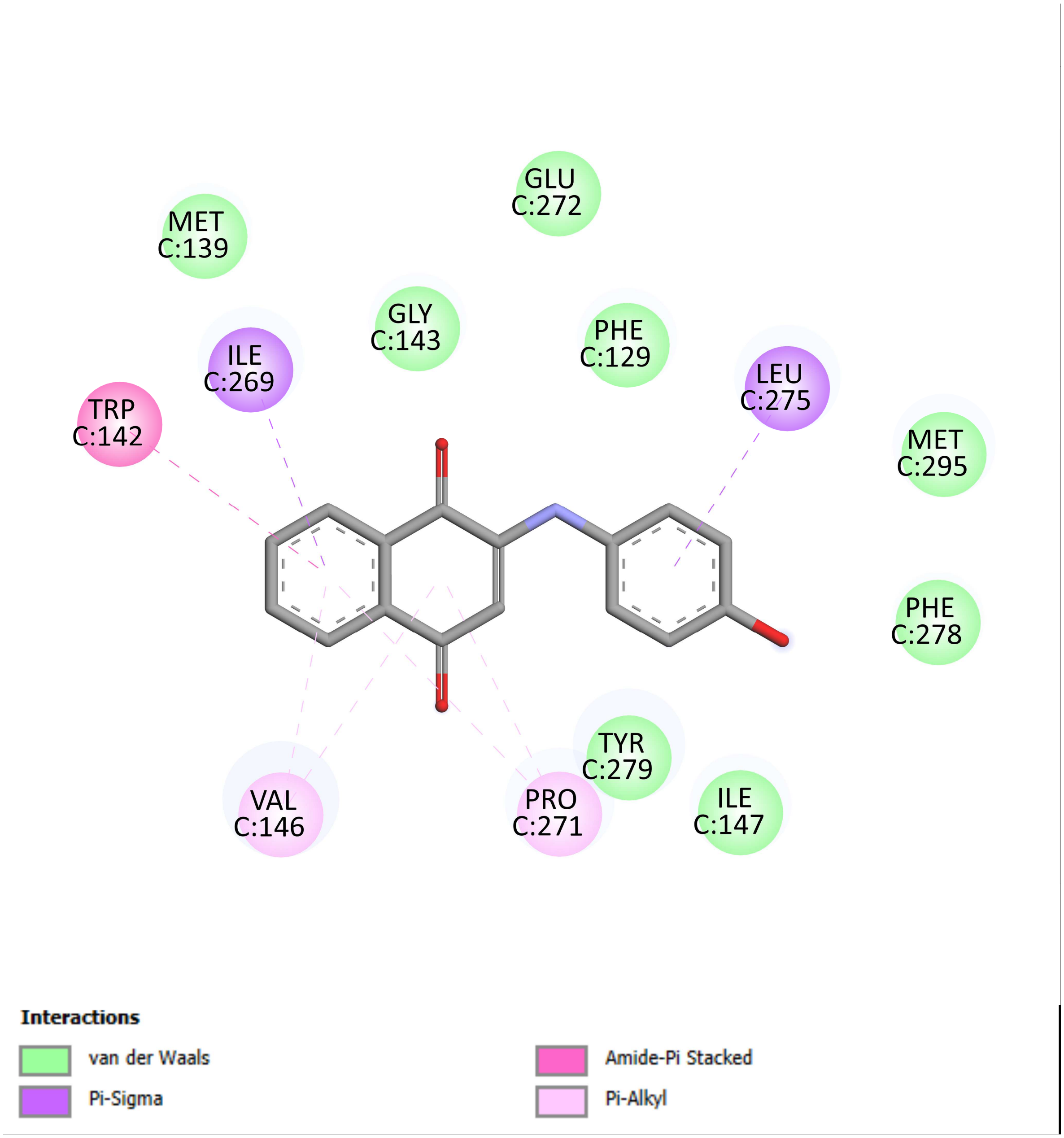

Figure S16. 2D representation of the interactions between compound 6 and residues of CYT BC1 protein (PDB ID: 4PD4). Hydrogen atoms have been omitted in some cases for clarity.

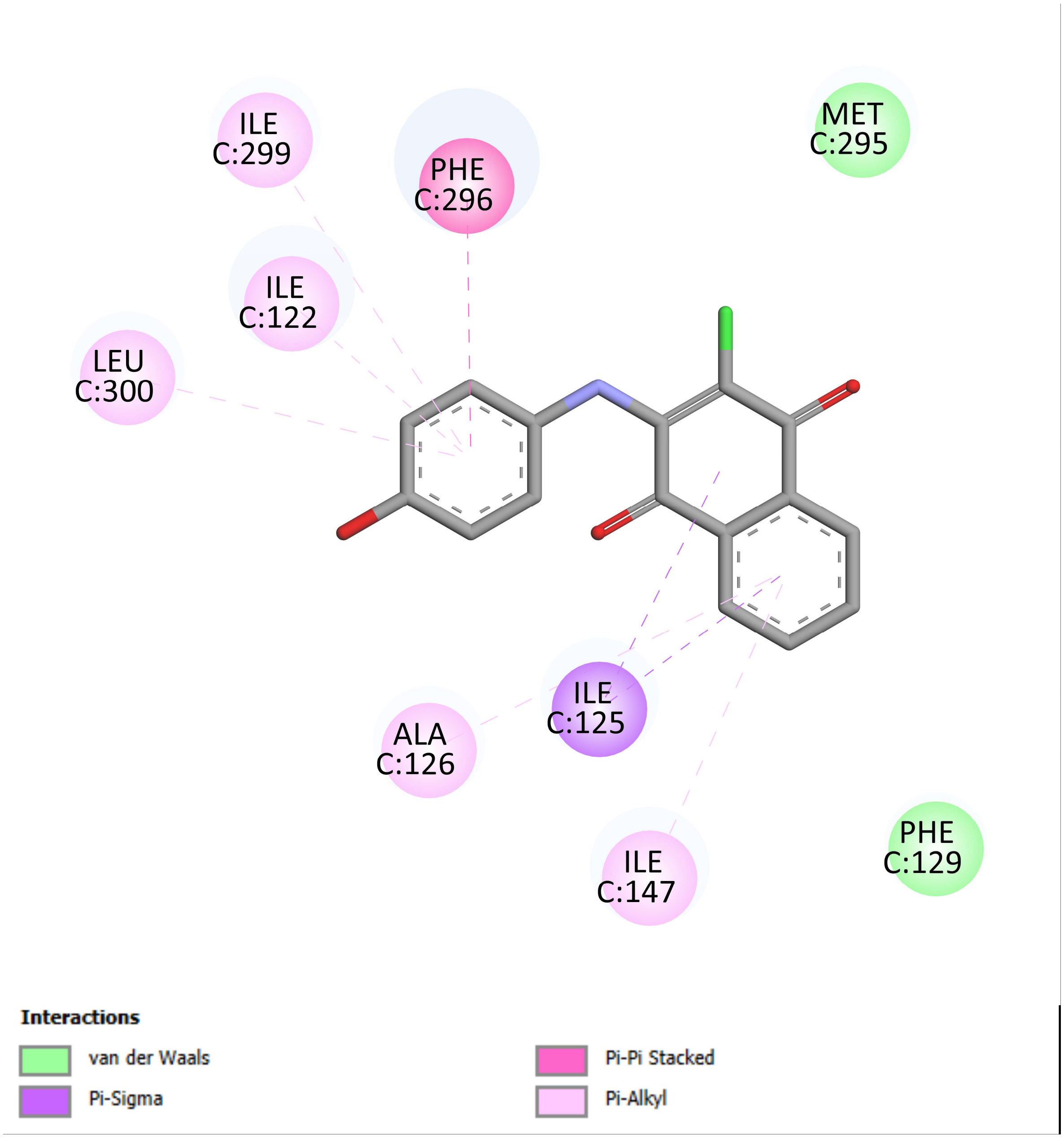

Figure S17. 2D representation of the interactions between compound 7 and residues of CYT BC1 protein (PDB ID: 4PD4). Hydrogen atoms have been omitted in some cases for clarity.

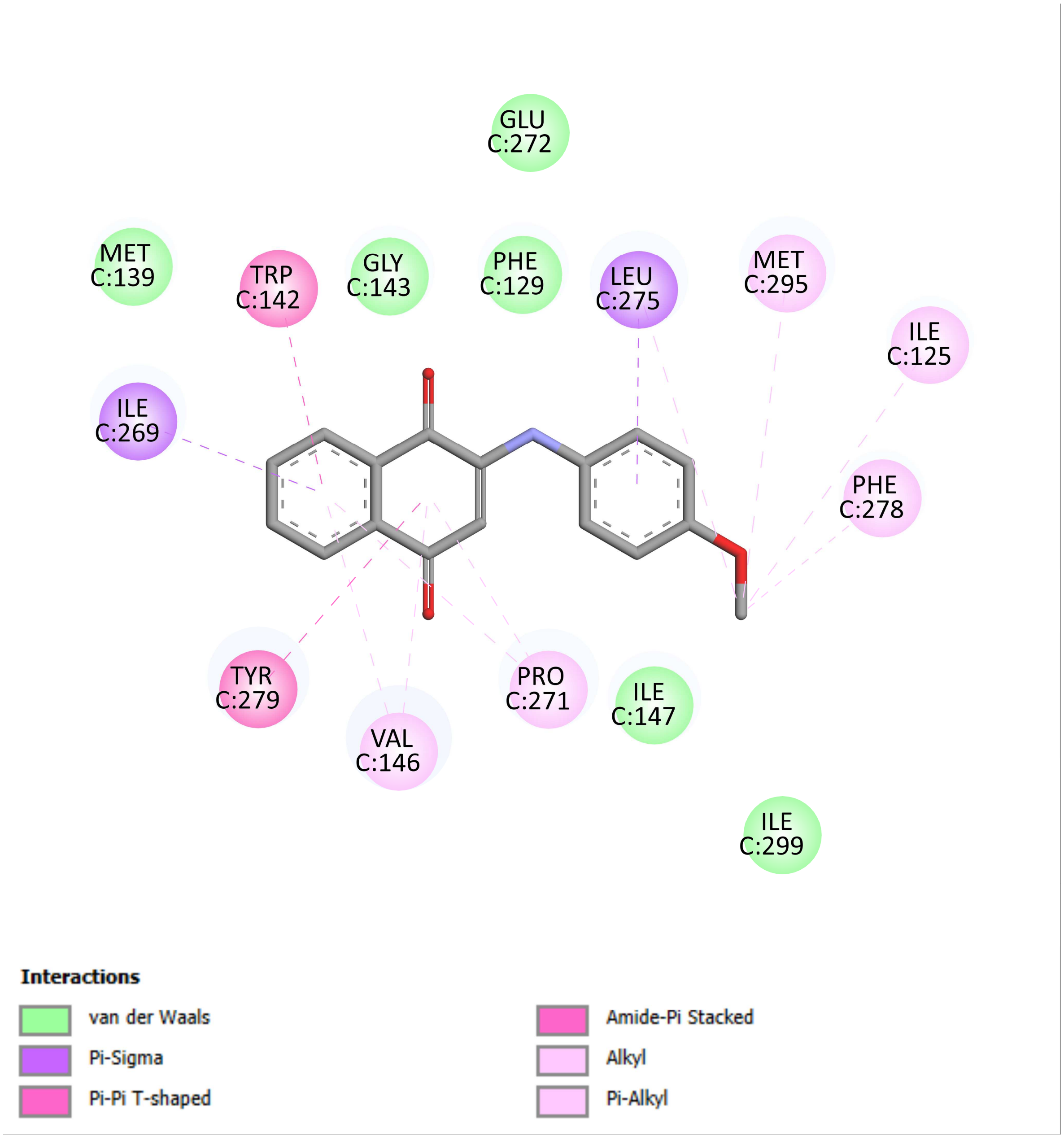

Figure S18. 2D representation of the interactions between compound 8 and residues of CYT BC1 protein (PDB ID: 4PD4). Hydrogen atoms have been omitted in some cases for clarity.

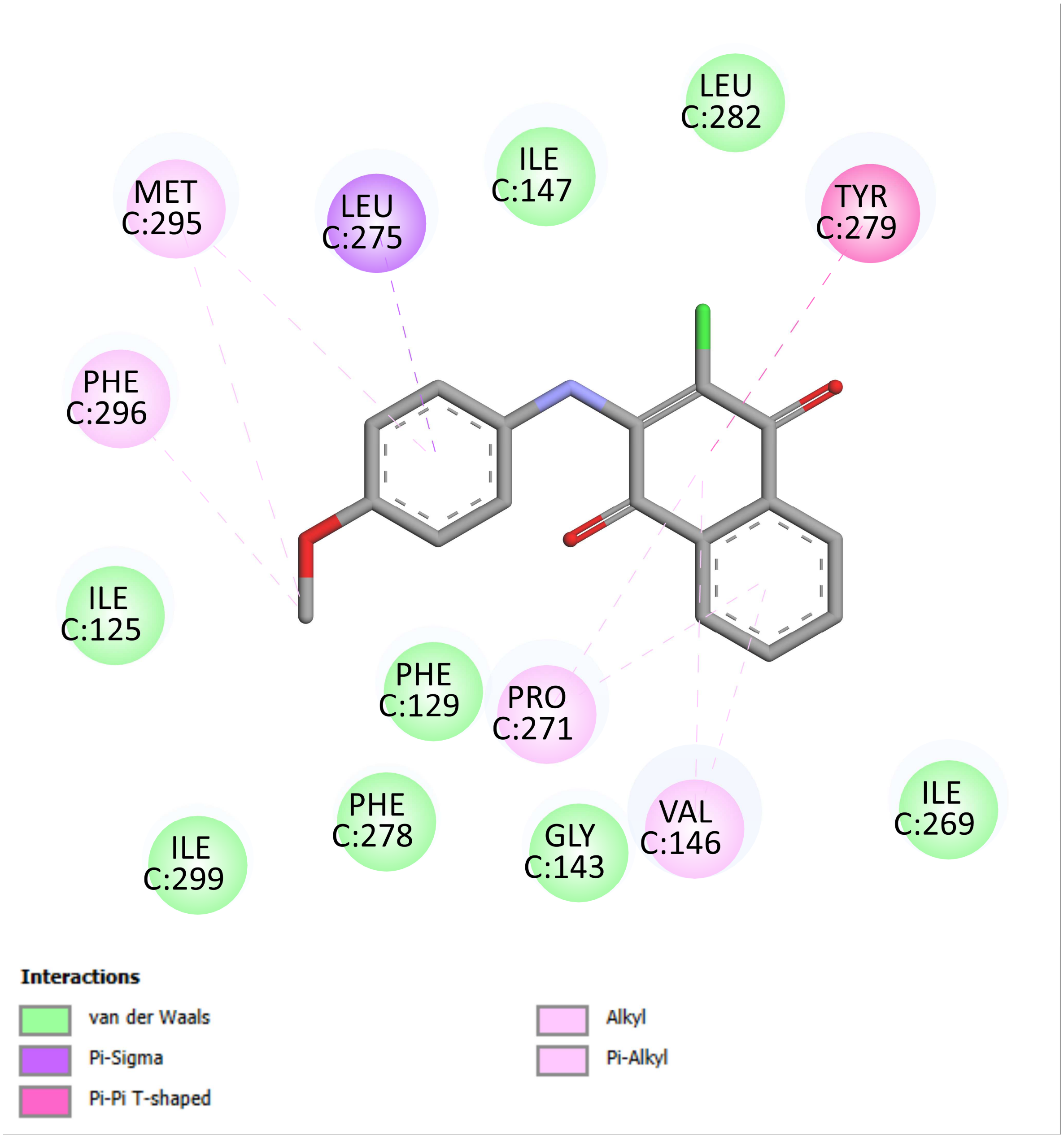

Figure S19. 2D representation of the interactions between compound 9 and residues of CYT BC1 protein (PDB ID: 4PD4). Hydrogen atoms have been omitted in some cases for clarity.

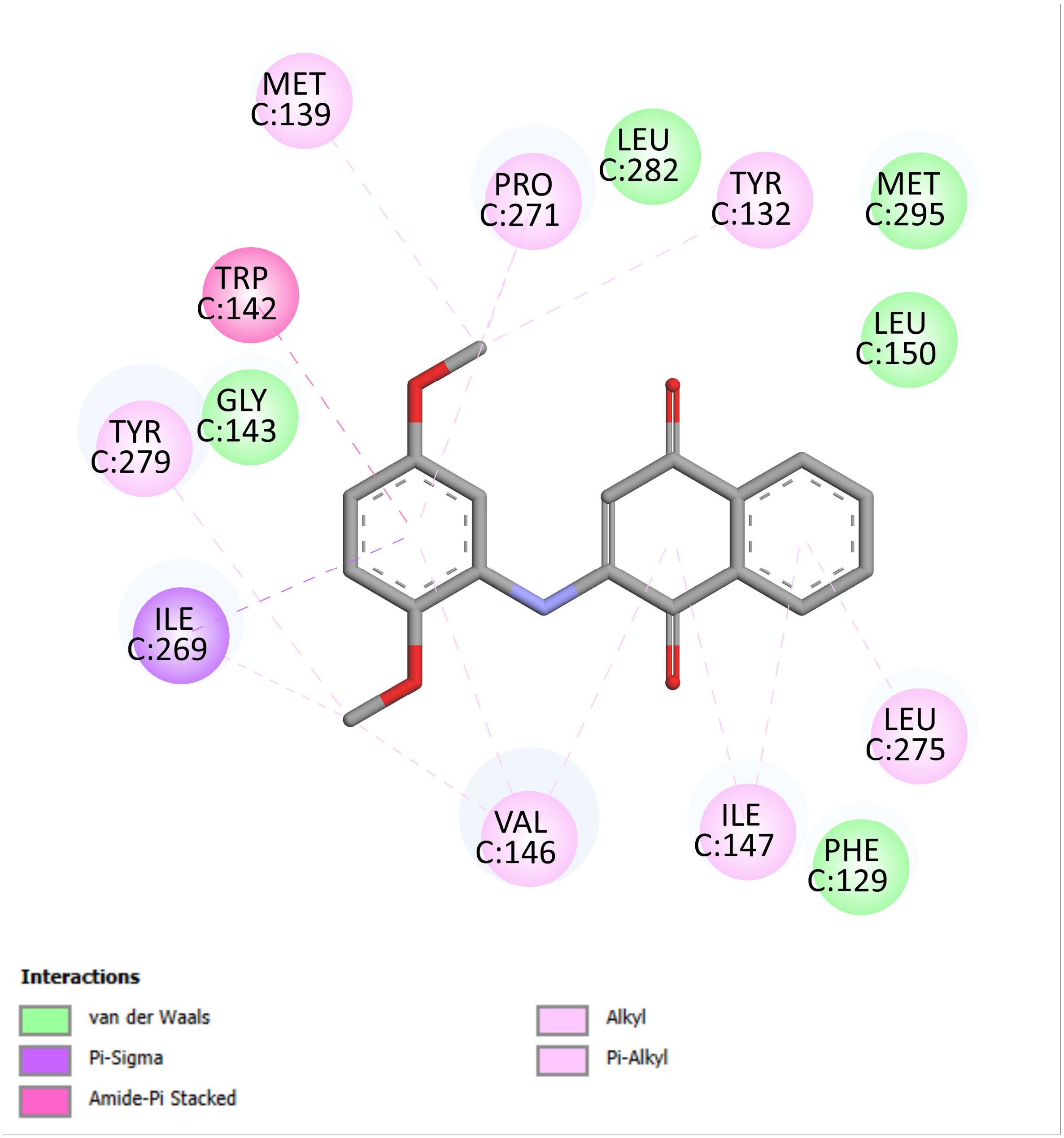

Figure S20. 2D representation of the interactions between compound 10 and residues of CYT BC1 protein (PDB ID: 4PD4). Hydrogen atoms have been omitted in some cases for clarity.

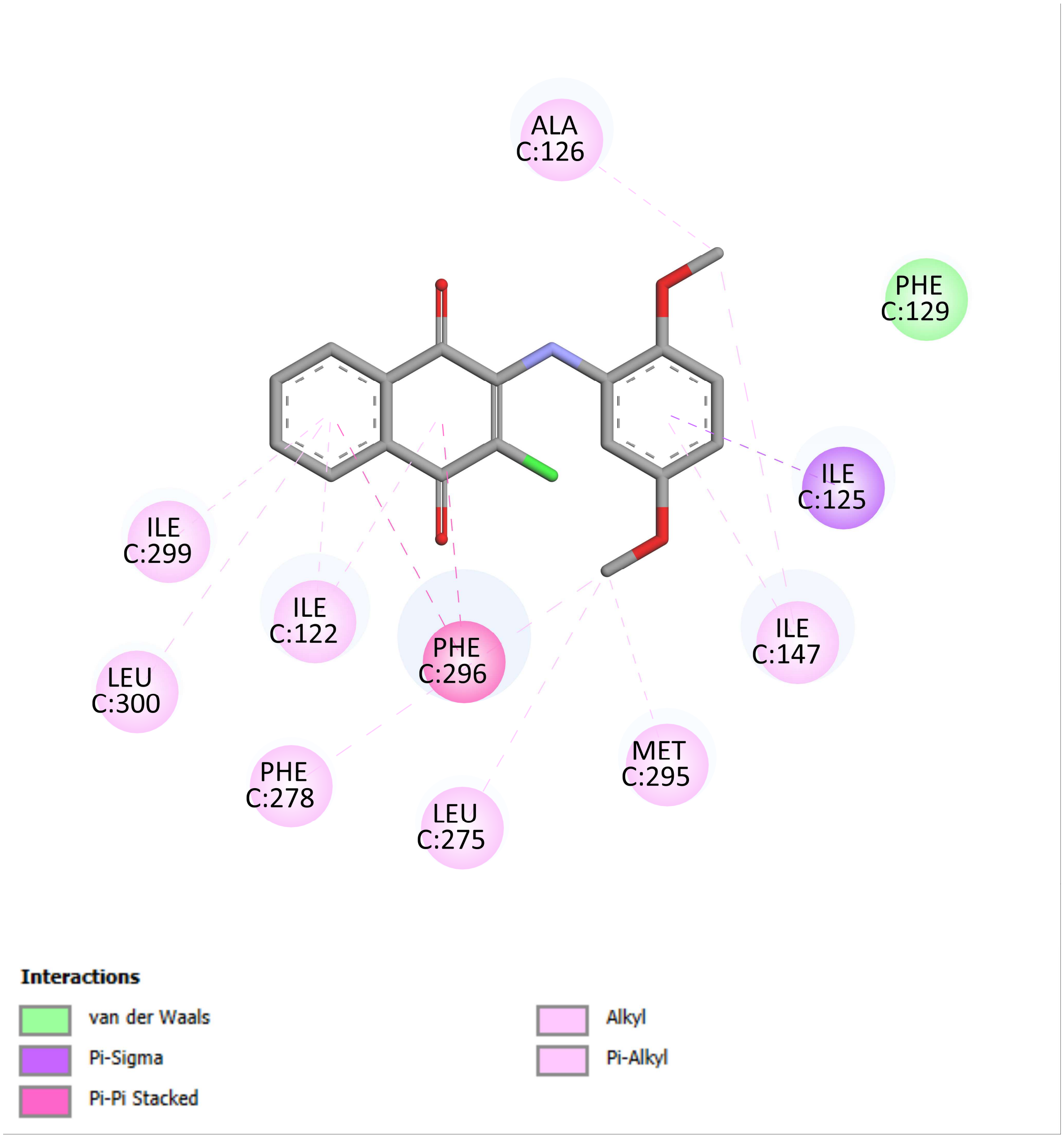

Figure S21. 2D representation of the interactions between compound 11 and residues of CYT BC1 protein (PDB ID: 4PD4). Hydrogen atoms have been omitted in some cases for clarity.

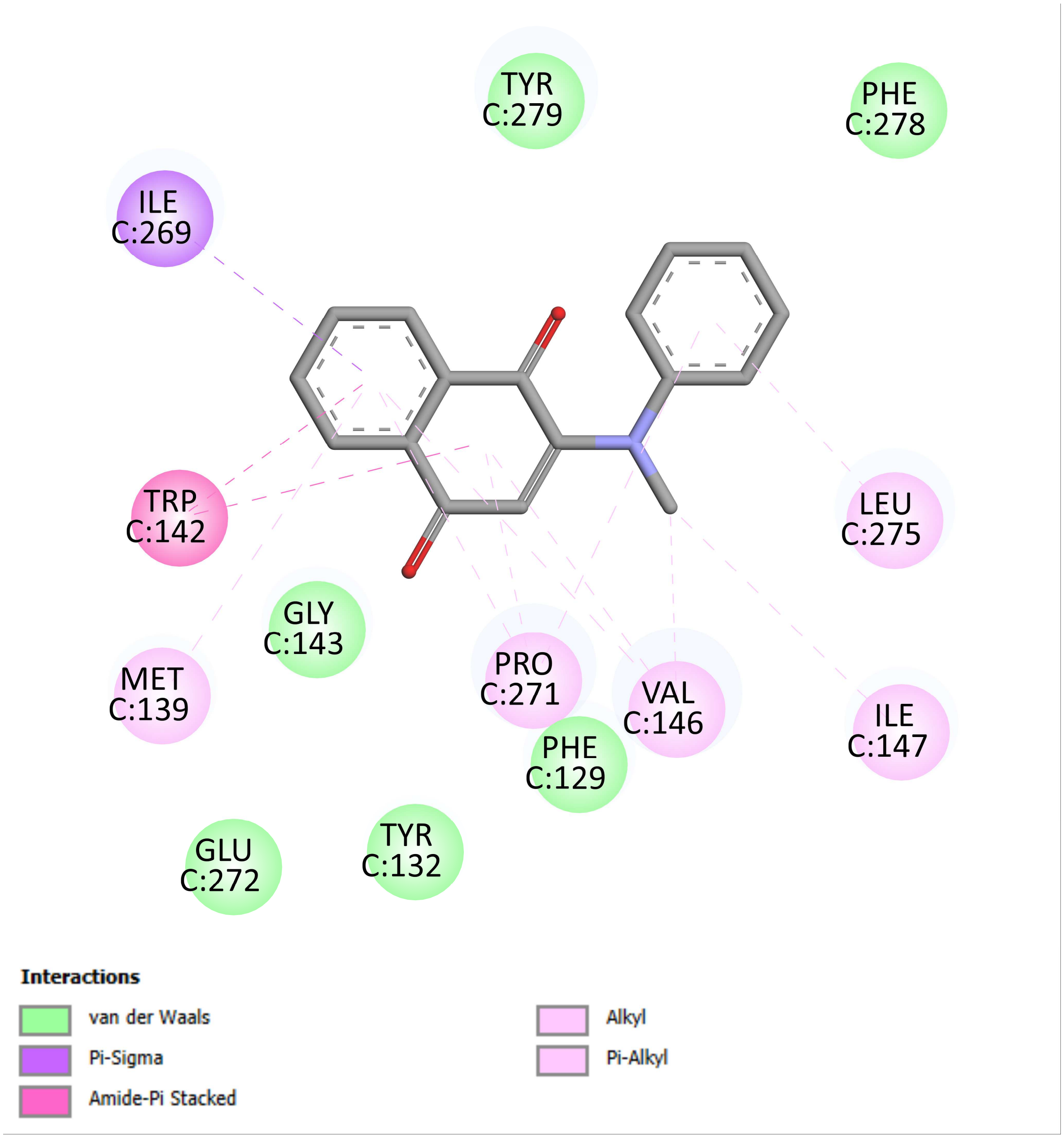

Figure S22. 2D representation of the interactions between compound 12 and residues of CYT BC1 protein (PDB ID: 4PD4). Hydrogen atoms have been omitted in some cases for clarity.

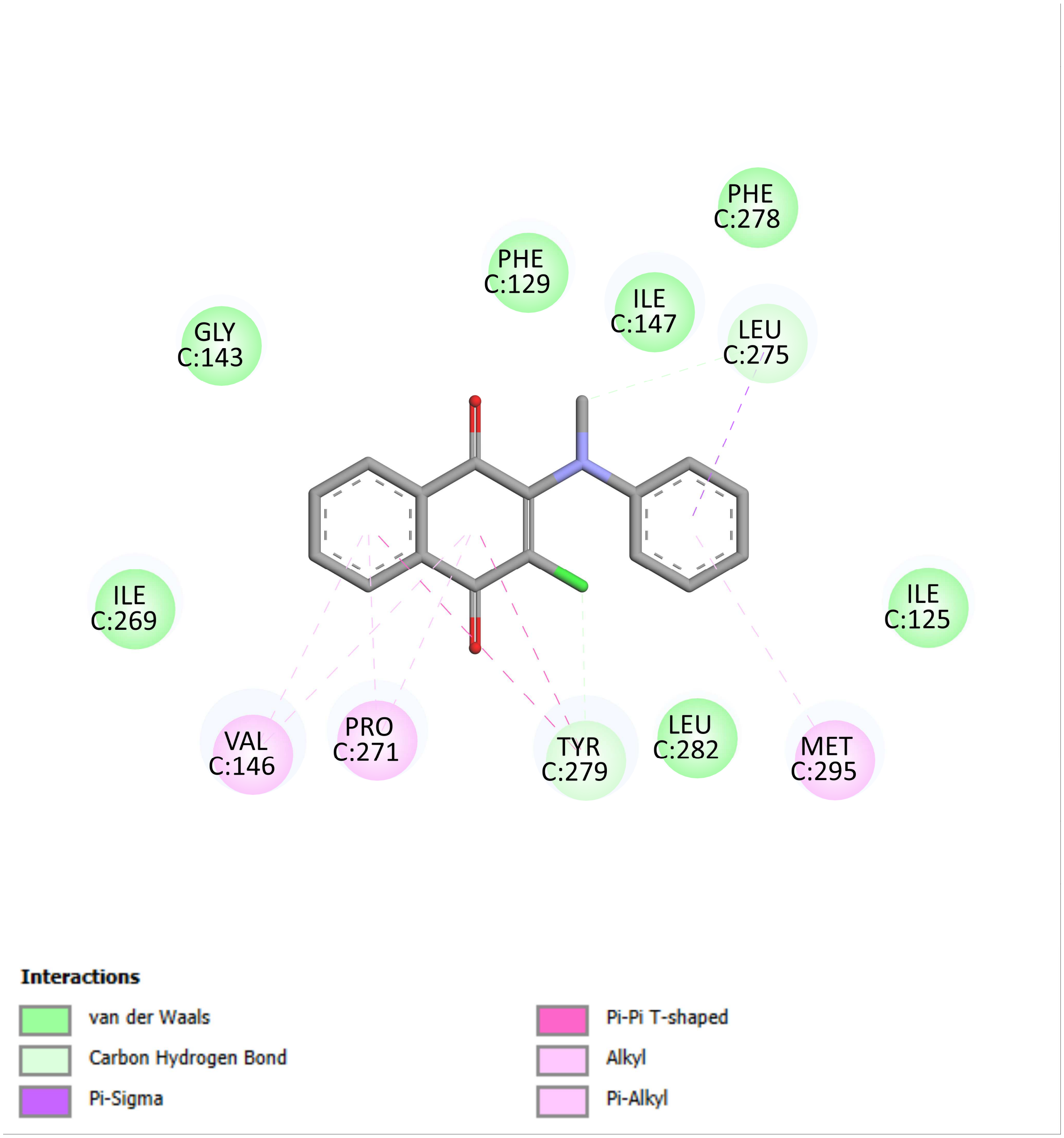

Figure S23. 2D representation of the interactions between compound 1 and residues of Plasmodium falciparum dihydroorotate dehydrogenase (PDB ID: 5FI8). Hydrogen atoms have been omitted in some cases for clarity.

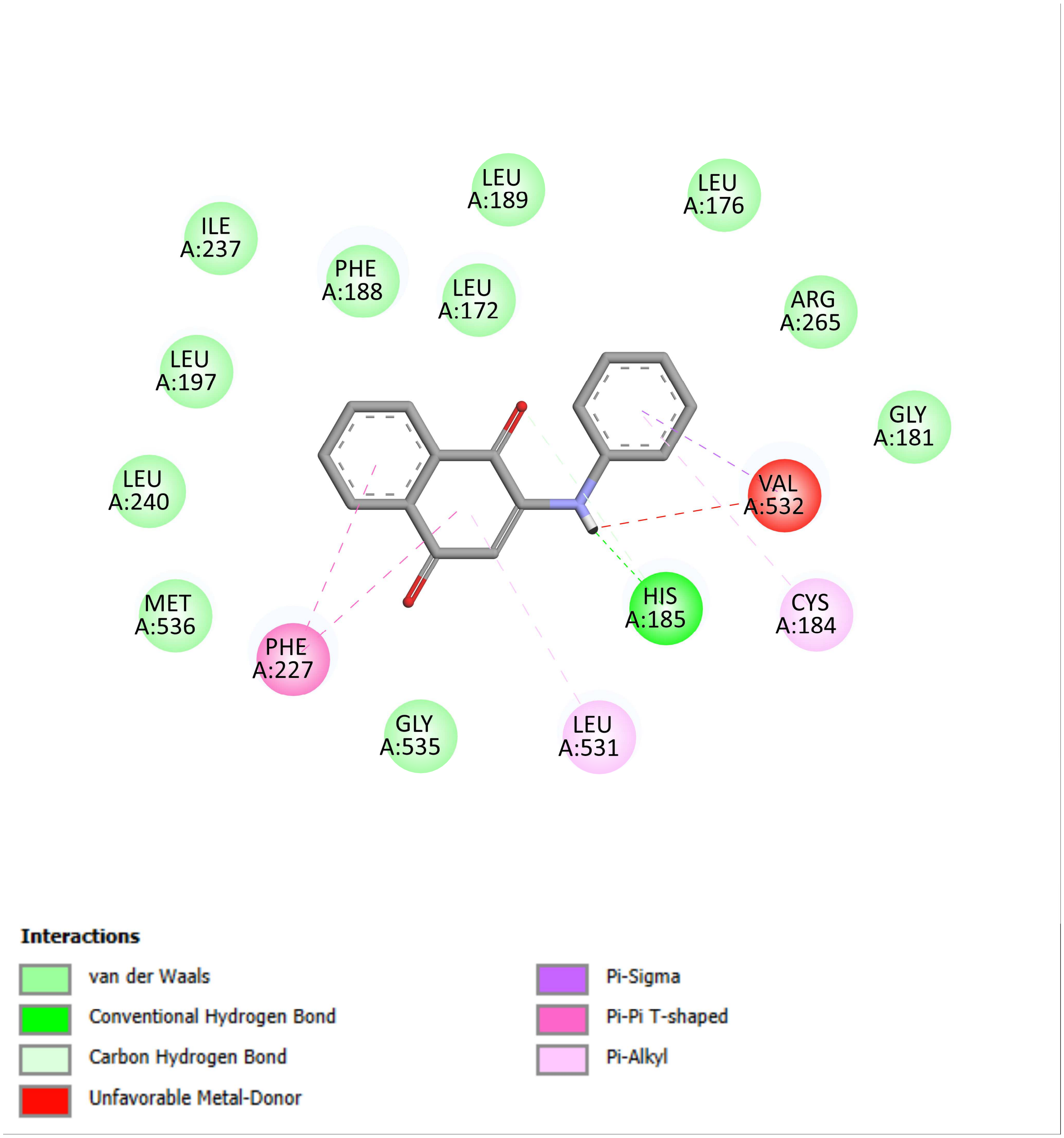

Figure S24. 2D representation of the interactions between compound 2 and residues of Plasmodium falciparum dihydroorotate dehydrogenase (PDB ID: 5FI8). Hydrogen atoms have been omitted in some cases for clarity.

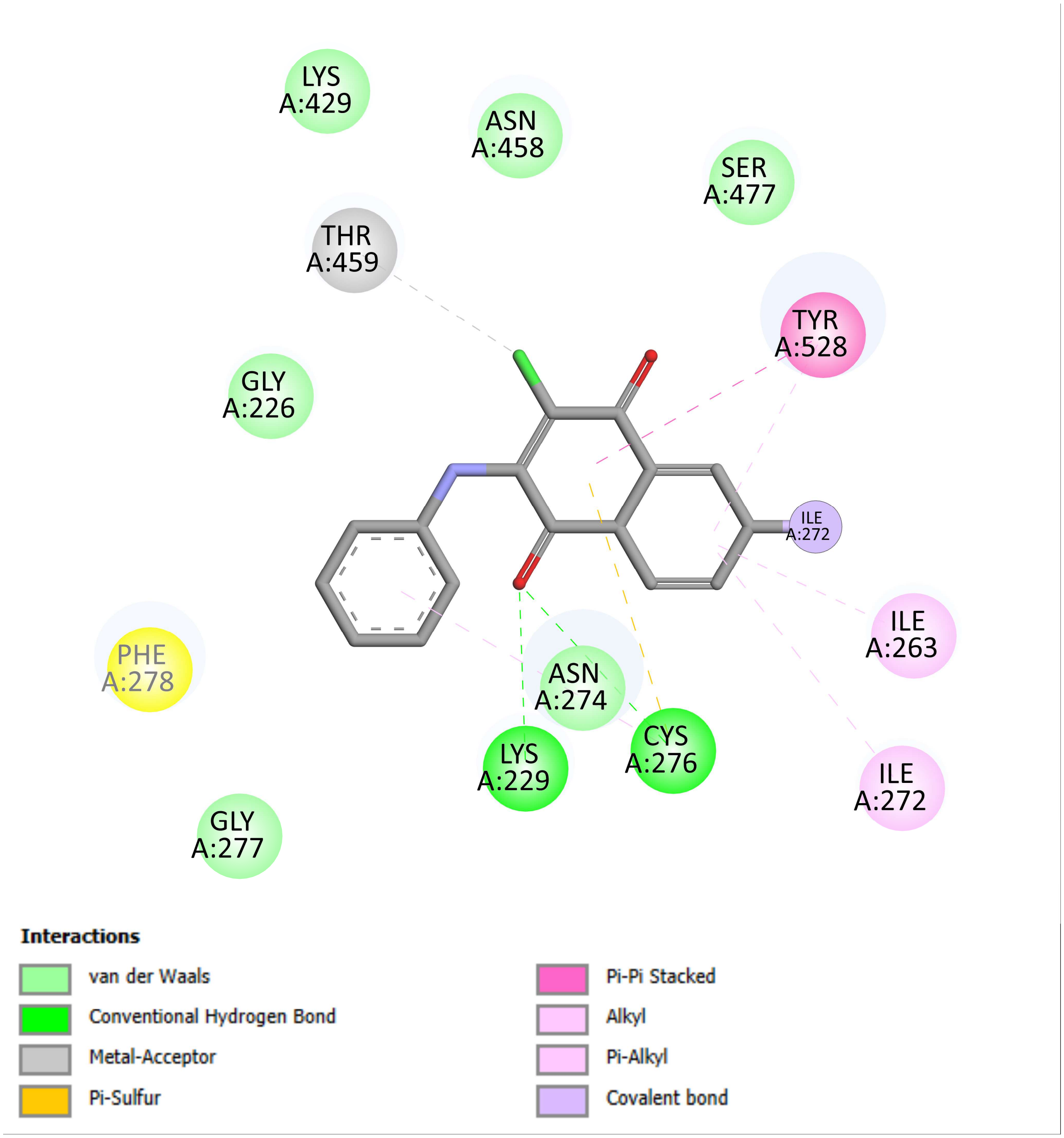

Figure S25. 2D representation of the interactions between compound 4 and residues of Plasmodium falciparum dihydroorotate dehydrogenase (PDB ID: 5FI8). Hydrogen atoms have been omitted in some cases for clarity.

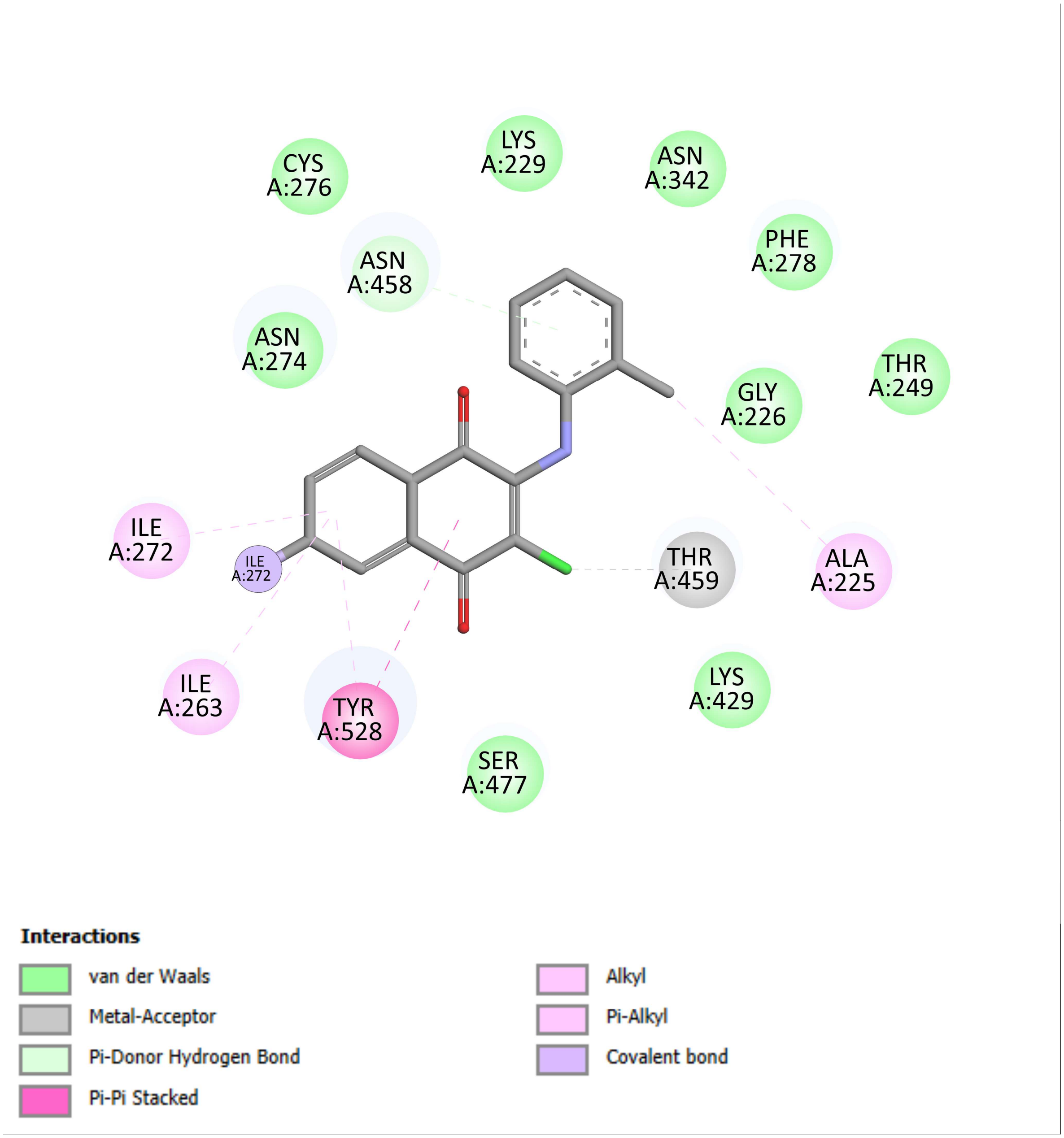

Figure S26. 2D representation of the interactions between compound 5 and residues of Plasmodium falciparum dihydroorotate dehydrogenase (PDB ID: 5FI8). Hydrogen atoms have been omitted in some cases for clarity.

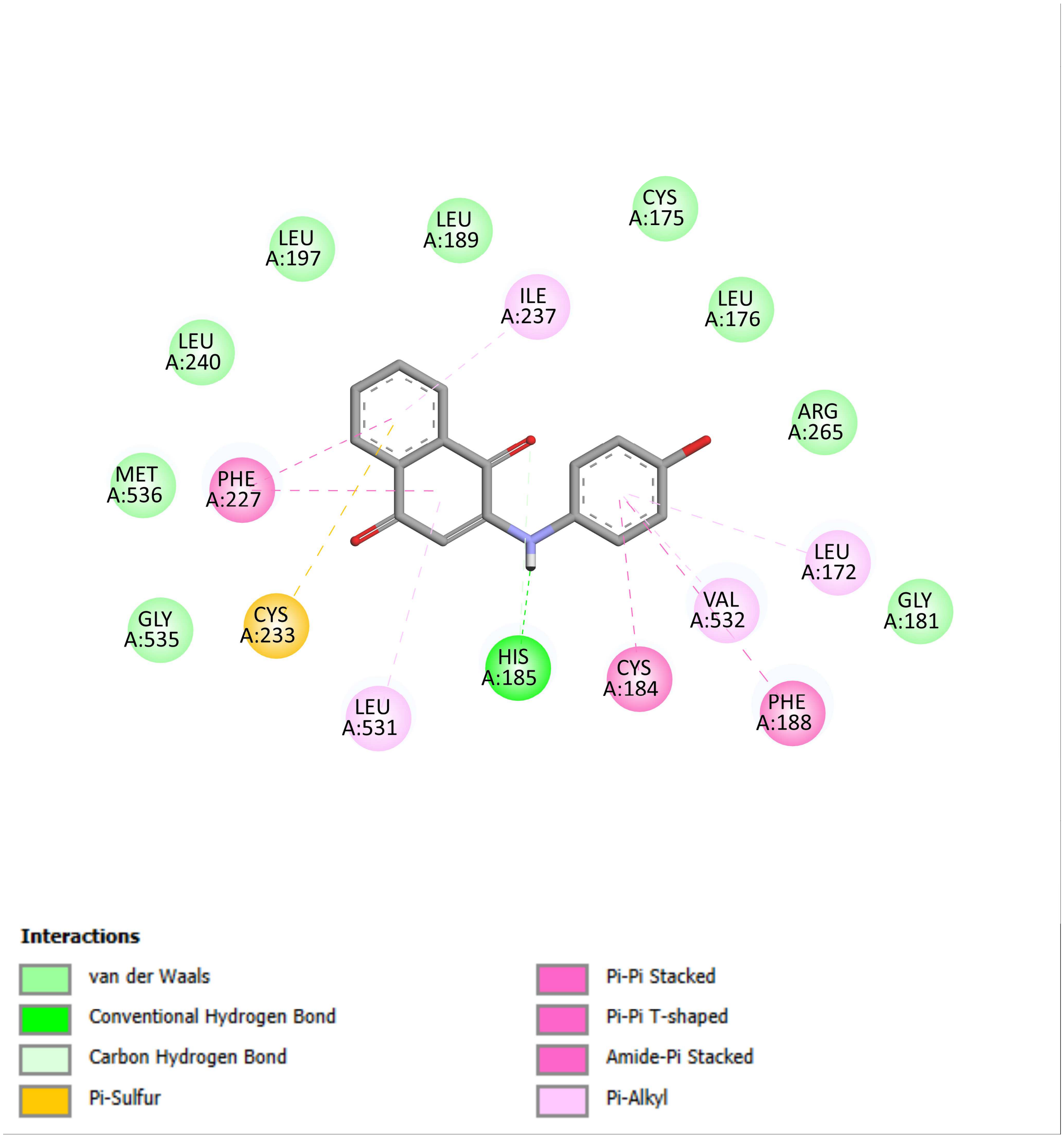

Figure S27. 2D representation of the interactions between compound 6 and residues of Plasmodium falciparum dihydroorotate dehydrogenase (PDB ID: 5FI8). Hydrogen atoms have been omitted in some cases for clarity.

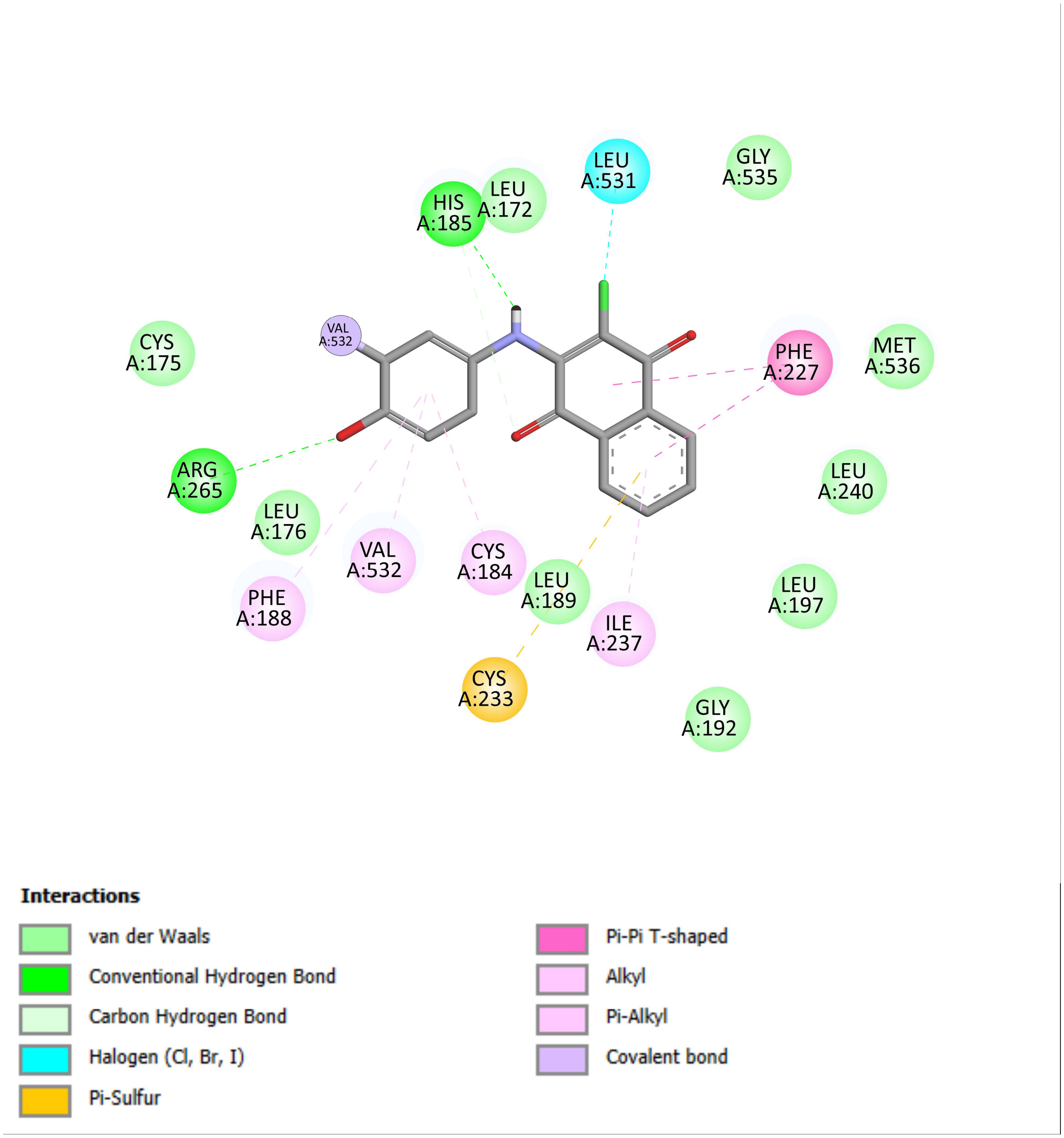

Figure S28. 2D representation of the interactions between compound 7 and residues of Plasmodium falciparum dihydroorotate dehydrogenase (PDB ID: 5FI8). Hydrogen atoms have been omitted in some cases for clarity.

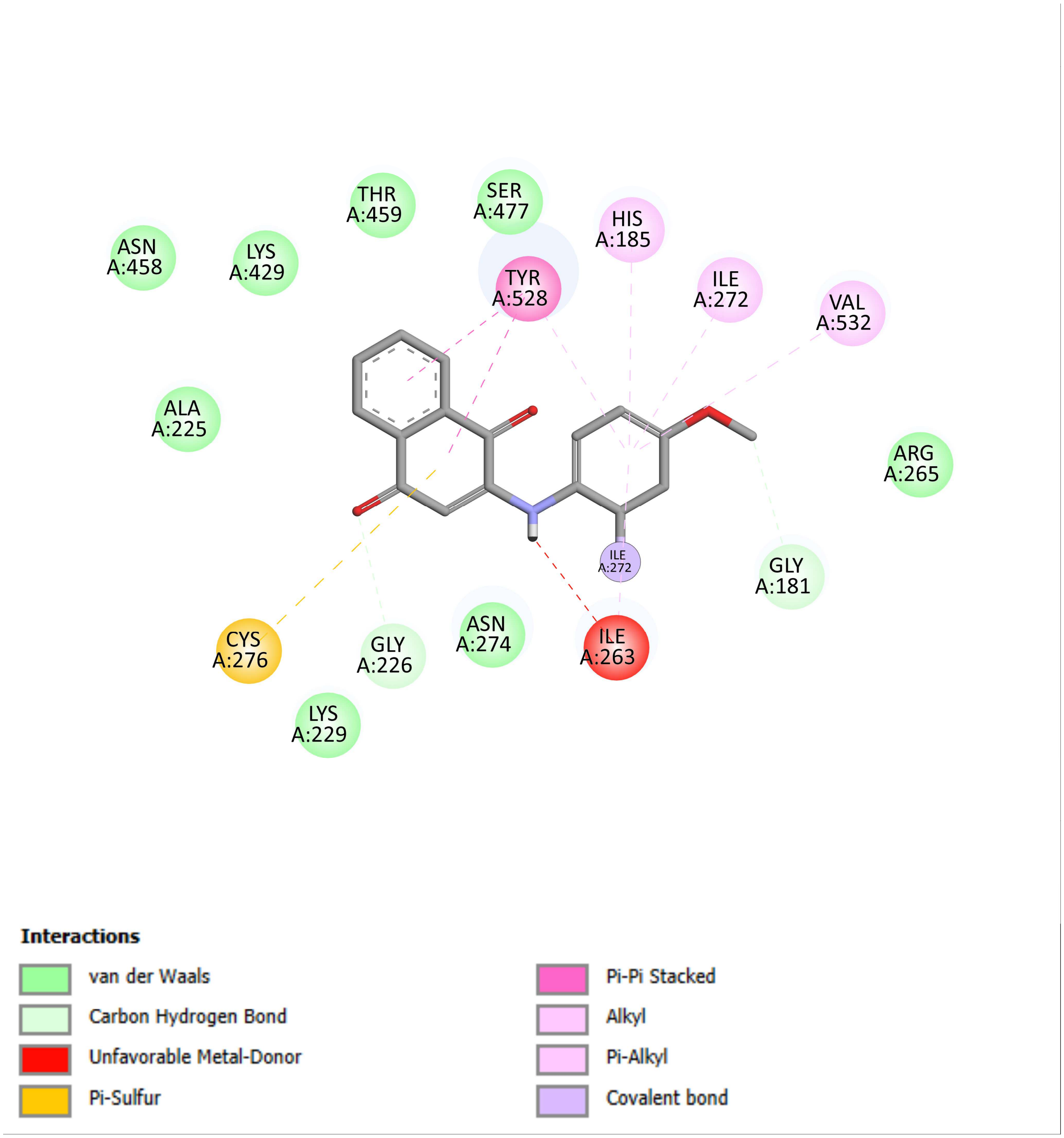

Figure S29. 2D representation of the interactions between compound 8 and residues of Plasmodium falciparum dihydroorotate dehydrogenase (PDB ID: 5FI8). Hydrogen atoms have been omitted in some cases for clarity.

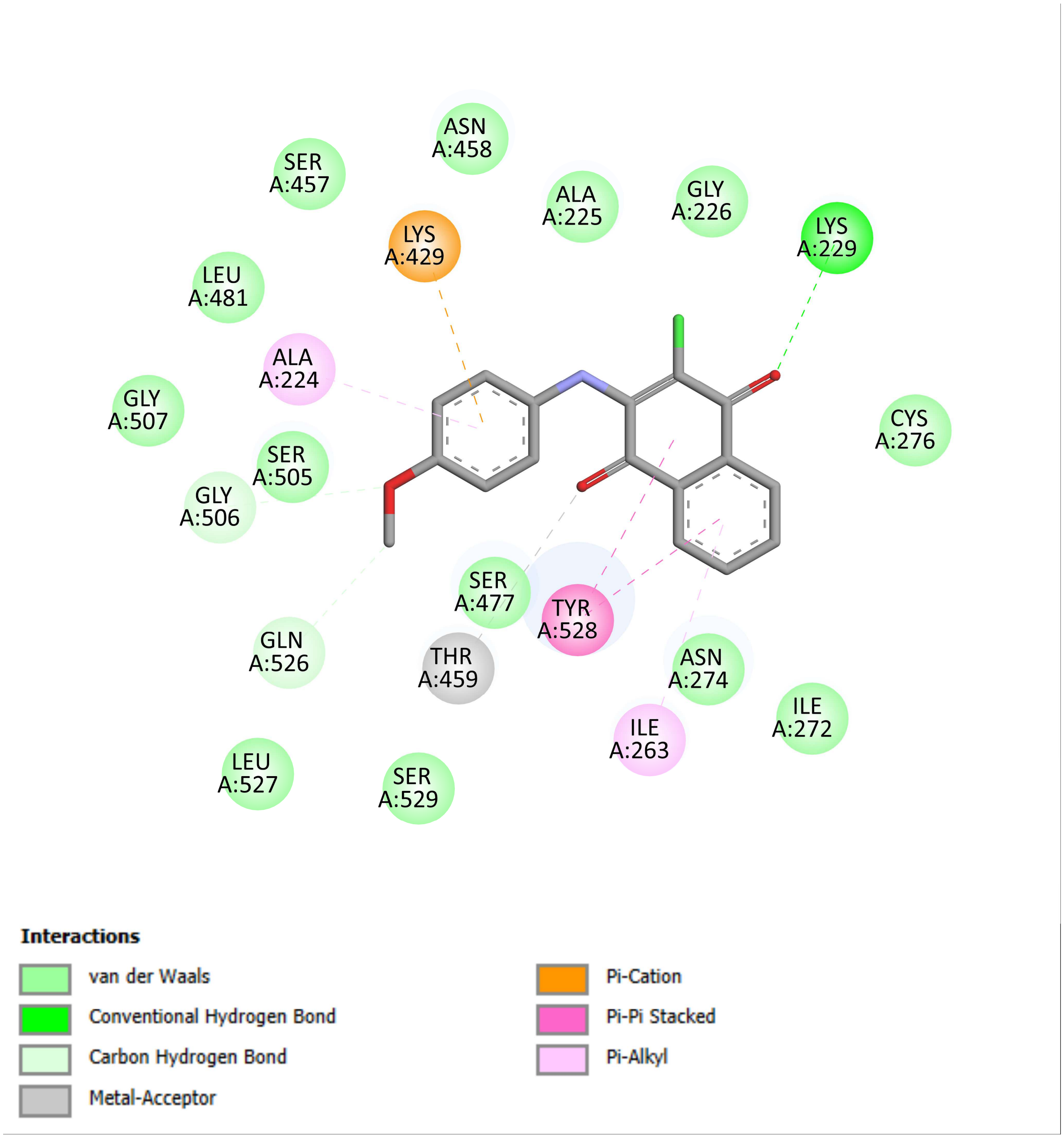

Figure S30. 2D representation of the interactions between compound 9 and residues of Plasmodium falciparum dihydroorotate dehydrogenase (PDB ID: 5FI8). Hydrogen atoms have been omitted in some cases for clarity.

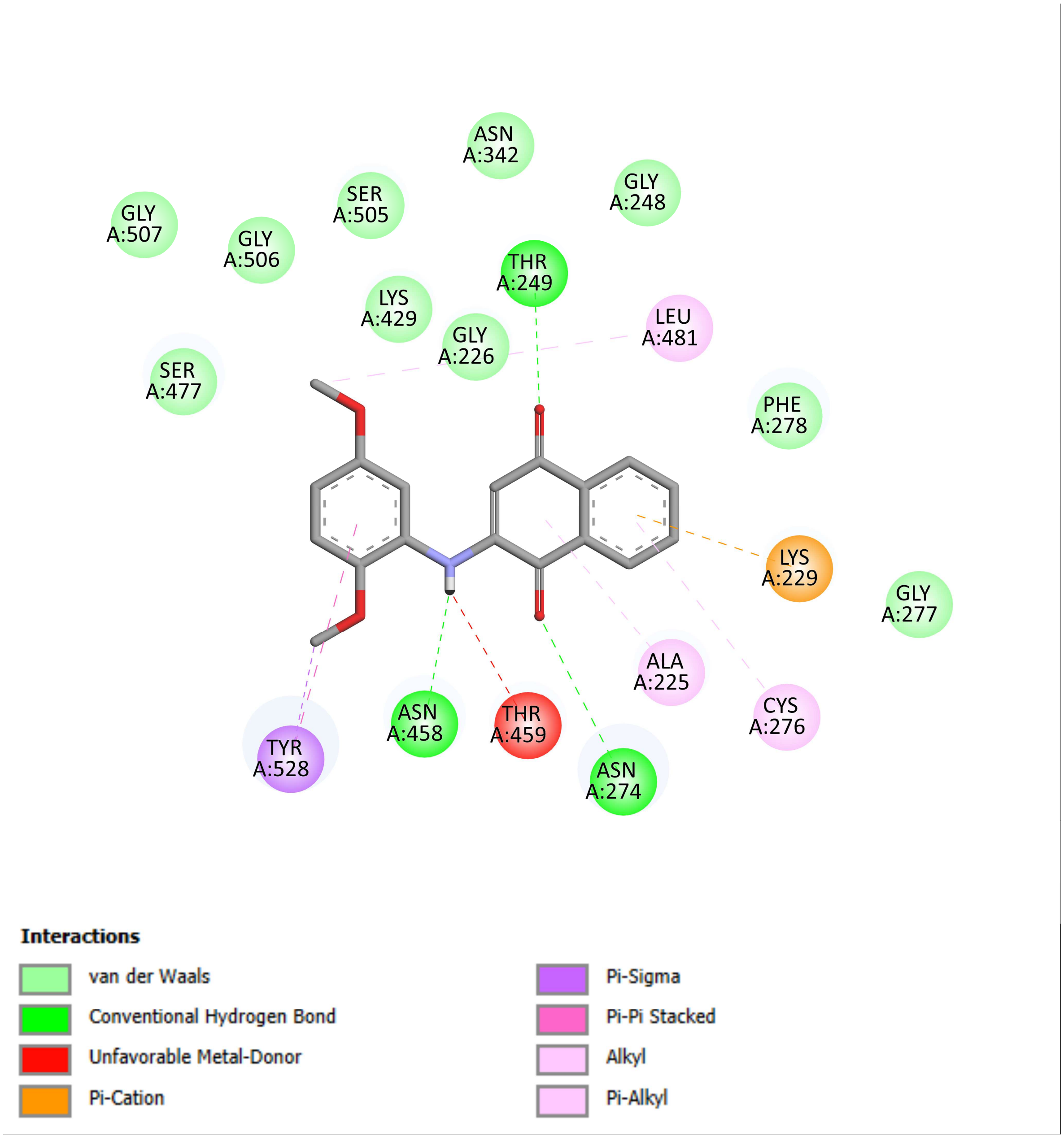

Figure S31. 2D representation of the interactions between compound 10 and residues of Plasmodium falciparum dihydroorotate dehydrogenase (PDB ID: 5FI8). Hydrogen atoms have been omitted in some cases for clarity.

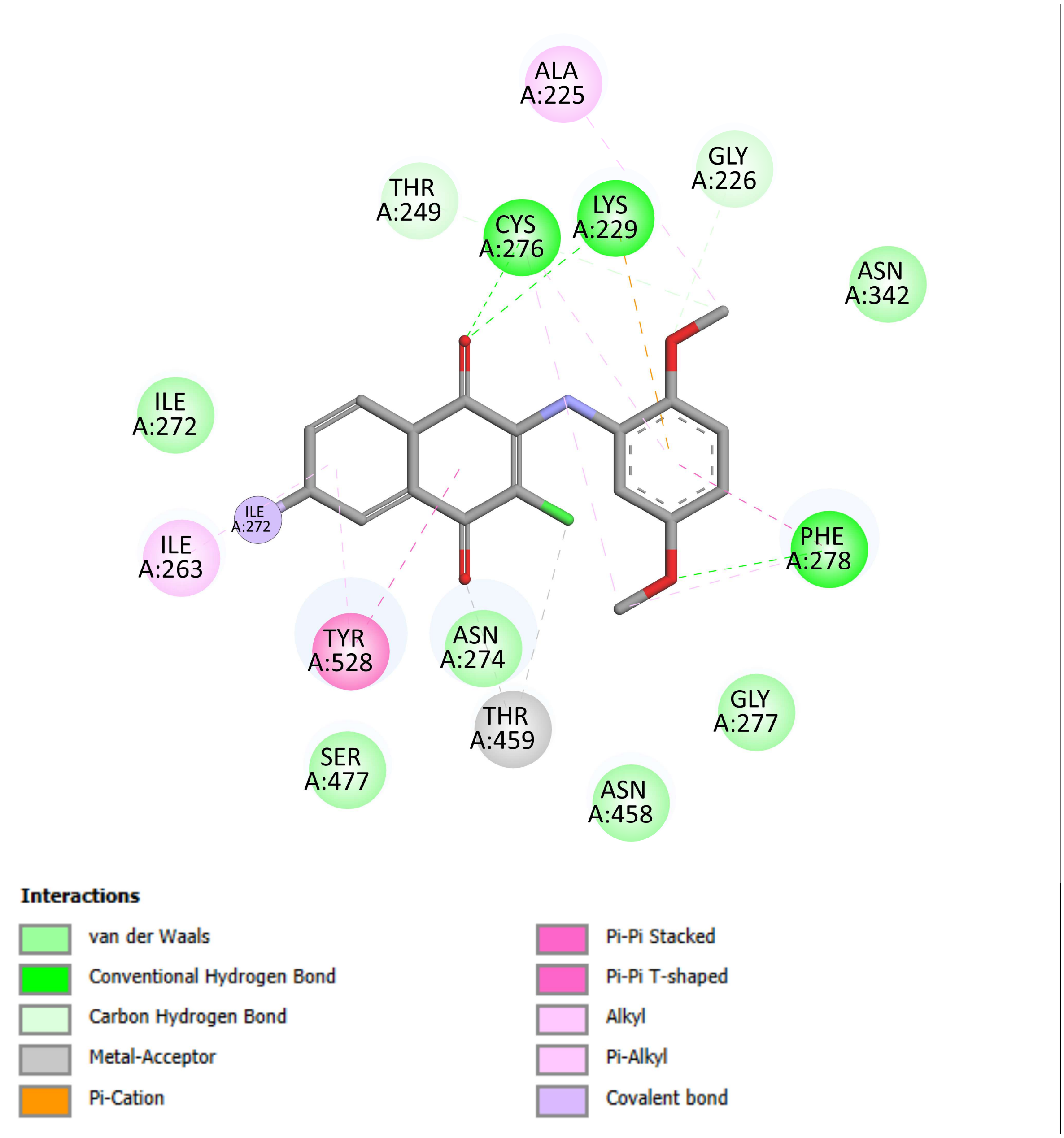

Figure S32. 2D representation of the interactions between compound 11 and residues of Plasmodium falciparum dihydroorotate dehydrogenase (PDB ID: 5FI8). Hydrogen atoms have been omitted in some cases for clarity.

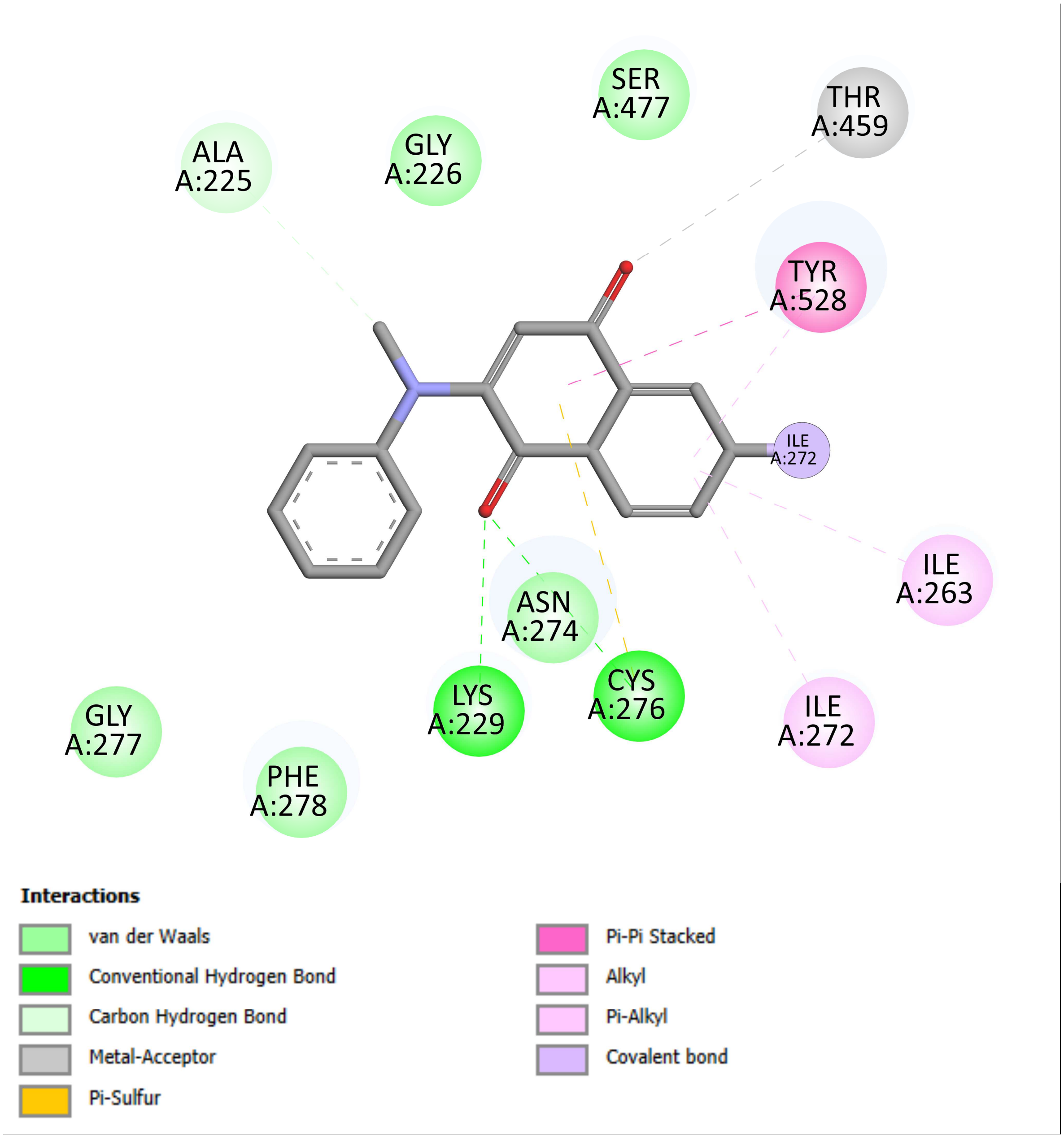

Figure S33. 2D representation of the interactions between compound 12 and residues of Plasmodium falciparum dihydroorotate dehydrogenase (PDB ID: 5FI8). Hydrogen atoms have been omitted in some cases for clarity.

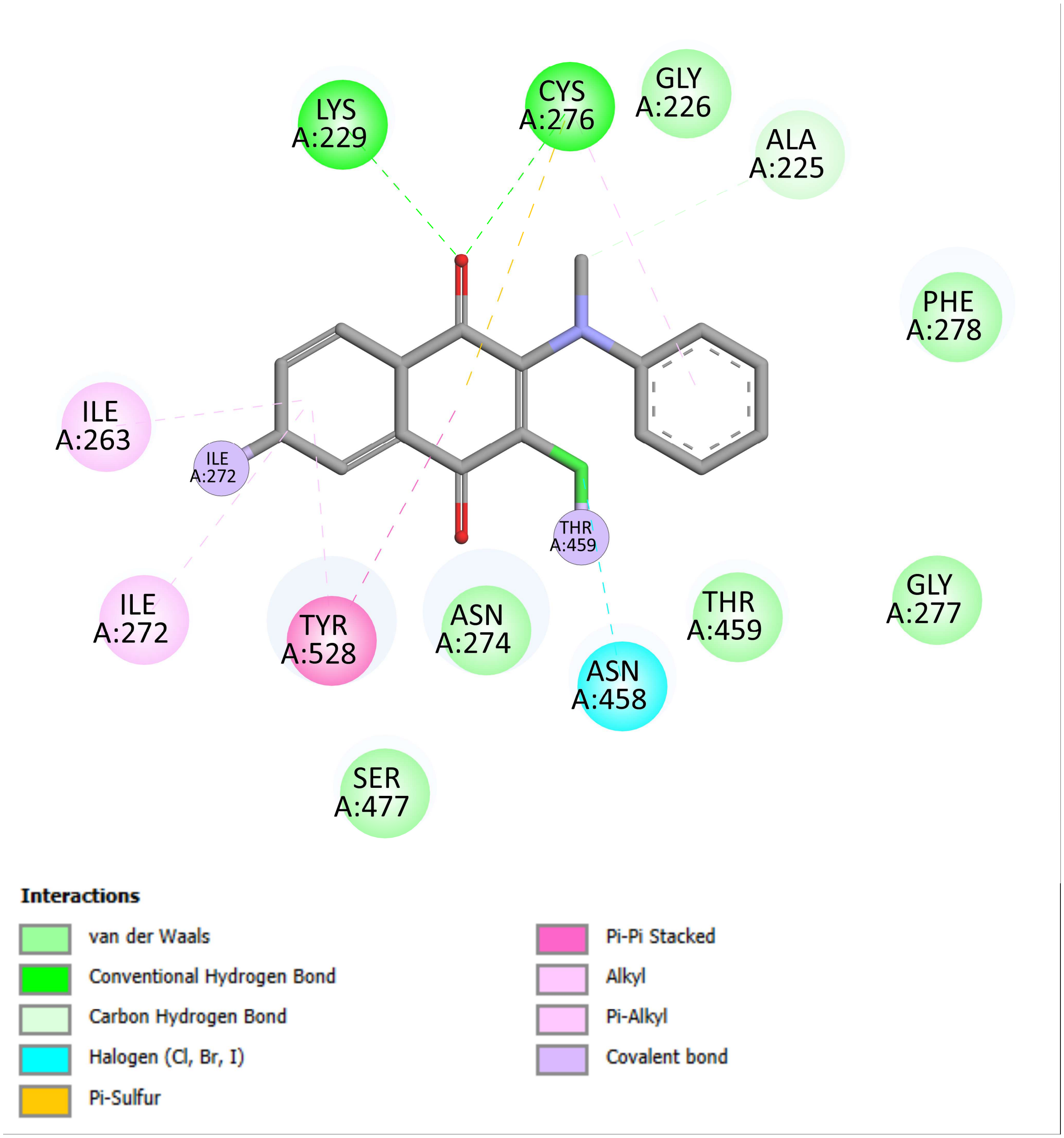

Supplement: Supplementary file 1 [file ijms-25-10670-s001.zip › ijms-3240793-supplementary.pdf]
